# Proteomic machine-learning signatures associated with carotid plaque: A population-based hypothesis-generating study

**DOI:** 10.1371/journal.pone.0358765

**Published:** 2026-09-24

**Authors:** Anna Szpakowicz, Wojciech Lesiński, Jakub K. Tuchliński, Urszula Roszkowska, Marlena Dubatówka, Zofia Stachurska, Marcin Kondraciuk, Witold R. Rudnicki, Karol A. Kamiński

**Affiliations:** 1 Department of Cardiology and Internal Medicine with Cardiac Intensive Care Unit, Medical University of Białystok, Białystok, Poland; 2 Faculty of Computer Science, University of Białystok, Białystok, Poland; 3 Department of Population Medicine and Lifestyle Diseases Prevention, Medical University of Białystok, Białystok, Poland; 4 Population Research Centre, Medical University of Białystok, Białystok, Poland; 5 Computing Centre, University of Białystok, Białystok, Poland; King Abdulaziz University, SAUDI ARABIA

## Abstract

Carotid plaque (CP) is a surrogate marker of cardiovascular disease, and high-risk morphology significantly increases the risk of stroke. The aim of this study was to develop advanced machine learning (ML) models to predict the presence of CP in the general population. The study involved 693 participants from the Polish Longitudinal University Study – Bialystok PLUS, a representative sample of the local population. The analysis used 460 OLINK protein biomarkers. The data were analysed using the BORUTA algorithm based on the random forest classifier method. We identified 42 significant biomarkers associated with CP, the most important of which were GDF-15, CDCP1, CHIT1, FLT3LG, CDH2 and CRTAC1. Interestingly, the top 6 biomarkers had a highly speculative or unresolved association with atherosclerosis. Next, we developed an ML-based CP fingerprint comprising 25 biomarkers, which showed good internal cross-validated discrimination in subjects aged 30–70 years. We also generated a network of interactions for the 42 biomarkers using STRING database modules, and performed a functional analysis to assess the biological significance of the interactions detected. These results are hypothesis-generating and require external, prospective validation to identify cause-and-effect relationships.

## 1. Introduction

According to the latest 2024 guidelines from the European Society of Cardiology, carotid atherosclerosis is classified into: stenosis below 50% - carotid plaque (CP) and stenosis ≥50% - carotid artery stenosis (CS) [1]. CP is a common condition. It is estimated that the prevalence of CPs in the world’s population of people aged 30–79, is 21.1% (816 million cases), CS 1.5% and significant thickening of the inner and middle membranes of the arteries (increased IMT) is 27.6% (1.1 billion cases). This prevalence of carotid plaque and stenosis increases consistently with age and is higher in men than in women [2]. In our population-based study (Białystok PLUS), the incidence of CPs was even higher- 46.2% of participants, whereas CS (≥50%) was found in 1.3% of cases (consistent with global data) [3]. The risk factors that have the greatest impact on the formation of atherosclerotic plaque include older age, male sex, smoking, and hypertension. Important factors also affecting the formation of carotid atherosclerosis are chronic hyperglycemia and hypercholesterolemia – especially considering the high concentration of low-density lipoprotein (LDL) [4].

Atherosclerosis is a pathological process with a multifaceted and complex mechanism. It can begin early in life, and research indicates that the presence of even insignificant carotid plaques can lead to life-threatening complications such as stroke or myocardial infarction [2,5,6]. The risk is particularly increased in the case of plaques with the so-called high-risk phenotype (especially in the presence of intraplaque hemorrhage). The main pathomechanisms of atherosclerosis are based on the initiation and amplification of a local inflammatory response involving immune cells, LDL, arterial epithelial cells, and inflammatory mediators [7]. These processes are not fully understood. In this context, advanced machine learning methods create an opportunity to analyze large databases, deepen and organize our knowledge.

The aim of this study was to develop an advanced ML model to predict the presence of carotid plaques in the general population based on clinical and proteomic data. The secondary aim was to identify the most important biomarkers associated with CP using ML. To this end, we used a protocol based on the application of feature selection (FS) methods for the identification of predictive features and an ML algorithm for model building, and the entire procedure was performed in cross-validation to verify the robustness of the models. Additionally, we sought to analyse the network of associations between various biomarkers by employing STRING database algorithms. In our previous publication, we used routine statistical tests to address these issues and used only one panel of biomarkers (cardiometabolic) [3]. For this project, we used alternative advanced analytical tools to gain a deeper description of the problem.

## 2. Materials

### 2.1 Study population and data collection

The data set used here is a part of the Bialystok PLUS cohort study, in which residents of Białystok aged 20–80 years were randomly selected from the Municipal Office database and invited to participate, constituting a representative sample of the local population [3]. They were recruited from 5 November 2018 to 24 March 2025. In this project, we used data from 698 consecutive participants.

### 2.2. Laboratory measurements

Fasting blood samples were collected from peripheral veins after at least 8 hours of fasting, and laboratory tests were performed immediately. Serum was obtained by centrifugation at 1810 G for 10 min at room temperature. Biochemical tests were performed using a Cobas c111 machine (Roche Diagnostics Ltd., Rotkreuz, Switzerland). Complete blood count was performed from blood collected in a tube with EDTA anticoagulant on a Mythic 18 analyzer (Cormay). Glycated haemoglobin A1c (HbA1c) levels were determined in blood drawn on EDTA using ion-exchange high-performance liquid chromatography on a D-10 device (Bio-Rad, Hercules, California, USA).

### 2.3. Carotid Doppler ultrasound examination

Carotid Doppler ultrasound examination was conducted with participants in the supine position using a Vivid™9 device equipped with a 9L-D linear array transducer with a frequency of 10 MHz (frequency range 4–11 MHz, GE Healthcare, Chicago, IL, USA). The assessment was made for the presence of CP in the following locations: left and right common carotid artery (CCA), left and right external carotid artery (ECA), left and right internal carotid artery (ICA), as well as left and right bifurcations (BIF).

Plaque assessment was standardised based on the Mannheim Intima-Media Thickness and Plaque Consensus [8], CP was defined as a local structure encroaching into the arterial lumen by at least 0.5 mm or 50% of the surrounding IMT, or having a thickness >1.5 mm measured from the media–adventitia interface to the intima–lumen interface. There were 3 ultrasound operators, they were blinded to clinical and proteomic data. Inter-rater reliability in the assessment of atherosclerotic plaques was evaluated in 100 individuals using Cohen’s kappa coefficient. High inter-rater reliability was demonstrated: between examiner 1 and 2 (κ = 0.786; 95% CI: 0.661–0.911; observed agreement: 90%; p < 0.001), between examiner 1 and 3 (κ = 0.696; 95% CI: 0.549–0.843; observed agreement: 86%; p < 0.001), and between examiner 2 and 3 (κ = 0.788; 95% CI: 0.665–0.911; observed agreement: 90%; p < 0.001). In addition, McNemar’s test did not reveal any significant systematic differences between the investigators in any of the comparisons (p = 0.344; p = 0.791; p = 0.754), indicating no significant asymmetry in the classification of the cases studied.

### 2.4. Clinical definitions

Blood pressure was measured in the sitting position after a minimum rest period of 5 minutes using the oscillometric method and an MG Comfort instrument (Omron Healthcare Co., Ltd., Terado-cho, Muko, Kyoto, Japan). Hypertension was defined based on participant-reported history; or use of antihypertensive drugs; or systolic blood pressure ≥ 140 mmHg; or diastolic blood pressure ≥ 90 mmHg.

Diabetes was defined as: fasting glucose concentration ≥ 126 mg/dL (7 mmol/L); glucose after 120 min of an oral glucose tolerance test (OGTT) ≥ 200 mg/dL (11.1 mmol/L); known history of diabetes; or use of antidiabetic drugs.

Hypercholesterolemia was defined as: total cholesterol concentration ≥ 190 mg/dL (4.9 mmol/L); low-density lipoprotein cholesterol (LDL-C) concentration ≥ 115 mg/dL (3.0 mmol/L); known history of hypercholesterolemia; or use of lipid-lowering drugs.

Obesity was defined as body mass index ≥ 30 kg/m². Smoking was defined as current smoking.

### 2.5. Proteomic analysis

Proteomic analysis was performed by the Olink Proteomics laboratory (Uppsala, Sweden). Serum samples were stored in a biobank at $-80^{\circ }$C, gradually warmed to $4^{\circ }$C for thawing, prepared, and then dispatched. We used cardiovascular, immune response, inflammatory, metabolic and cardiometabolic panels, which makes a total of 460 biomarkers. Biomarker levels were assessed through the proximity extension assay (PEA) multiplexing technique, which is based on quantitative PCR. Instead of absolute values, this method allows estimating the relative level of biomarkers expressed in the NPX unit (Normalised Protein eXpression) on a log_2_ scale. We decided not to remove from the analysis biomarkers with a high percentage of samples with measurements below the limit of detection (LOD) in order to avoid data leakage and overestimation of models. The proteomic data were not subjected to filtering or preprocessing. Data median imputation, normalization, feature selection, and model building were performed within each training fold. All analysed biomarkers are listed in S1 Table (eight biomarkers were duplicated across the panels).

## 3. Methods

### 3.1. Feature selection

Feature selection is an important step in the development and interpretation of machine learning (ML) models. Classifiers achieve the best accuracy when all used variables are informative. This is especially important when the number of variables is very large for two reasons. First, the computational complexity of model development can grow quickly with increasing dimensionality. Second, the quality of the final model can be significantly degraded when a large number of irrelevant or redundant variables are used.

There are three main classes of feature selection algorithms: filters, wrappers, and embedded algorithms. Filters are usually applied to each variable individually to check whether knowledge about the predictor improves knowledge about the decision variable. Wrappers utilise an ML algorithm that returns an estimate of feature importance. Embedded algorithms perform feature selection as part of the model building process.

Another classification of feature selection algorithms is based on their purpose. Minimal-optimal algorithms aim to find the minimal set of variables sufficient for building good predictive models. All-relevant algorithms aim to find all variables that are meaningfully connected with the decision variable.

Minimal-optimal algorithms are very useful for reducing computational complexity. However, this comes at a price. First, they tend to be very unstable when multiple highly correlated informative variables exist in the system. Second, they are less suitable when a deeper understanding of the system under scrutiny is the primary goal, as opposed to pure classifier performance. All-relevant algorithms are better suited in this case, since they provide all available information about the system.

Therefore, in the current study, we used two all-relevant feature selection algorithms: one wrapper and one filter.

#### 3.1.1. Boruta.

The primary feature selection algorithm used in this study was Boruta [9,10]. It is a wrapper built around the Random Forest (RF) classification algorithm [11]. It extends the system by adding randomised copies of the original variables, the so-called shadow or contrast variables, and builds an RF model on such an extended set of variables. It then compares the permutation importance of the original variables returned by RF with the importance of the most important shadow variable (MISV). The procedure is repeated multiple times, and then a statistical test is performed for each original variable to determine whether its importance is higher than the importance of the MISV. Variables that are statistically significantly more important than the MISV are deemed relevant.

#### 3.1.2. Multidimensional Feature Selector (MDFS).

The second feature selection algorithm used in the study was the Multidimensional Feature Selector (MDFS) [12,13]. This algorithm is a filter based on information theory and can discover variables that are related to the decision variable in a non-linear way, as well as in synergistic interactions with other variables. Similarly to Boruta, it utilises non-informative contrast variables to generate a null distribution of importance for non-informative variables. It was used in the study as an alternative feature selection algorithm for verification of the results.

### 3.2. Modelling

Models were obtained using the Random Forest (RF) classification algorithm [11]. RF is an ensemble learning algorithm that uses a collection of decision trees to make predictions. Each decision tree is built using a different bootstrap sample of the data set. Moreover, each split in the tree is obtained using the best possible split generated only for a randomly selected subset of variables. Each tree in the RF then casts a vote to produce a prediction.

Its ease of use and flexibility have fuelled its adoption, as it handles both classification and regression problems. RF also has an internal unbiased estimate of error, obtained by measuring the prediction error on out-of-bag (OOB) objects: each tree casts a vote for all objects that were not used for its generation (OOB objects), and the decision is compared with the true value. This method works well out of the box on most data sets [14]. RF were used in two ways: for OOB predictions and in the cross-validation procedure.

A very important property of RF is its robustness in the presence of multiple correlated predictor variables. Thanks to the strong randomisation of individual trees in the ensemble, adding additional correlated predictors can improve model quality through noise reduction.

### 3.3. Cross-validation

Machine learning methods frequently generate models that are overfitted to the training set. In particular, the selection of variables used for modelling can result in strong overfitting. Due to its construction, the RF classifier is generally robust and not prone to overfitting, particularly when multiple variables carry a similar signal. Nevertheless, the influence of this effect was estimated by performing a cross-validation procedure.

In each repeat, the data set was split randomly into five parts in a stratified manner. Five models were then developed for each split, with four parts used as a training set and one as a validation set. Feature selection and data imputation were performed within each cross-validation fold. The entire procedure was repeated ten times to estimate the variance of the models due to sampling.

A single iteration of the protocol consisted of the following steps:

- Split the data into training and validation sets;
- Identify informative variables in the training set;
- Select the most informative variables;
- Build a model on the training set;
- Estimate the model on the validation set.

### 3.4. Evaluation metrics

Two measures were used to evaluate the obtained models. The area under the receiver operating characteristic curve (AUC) is a global measure of performance that can be applied to any classifier that ranks binary predictions for objects. The AUC measures how well the order of cases returned by classifiers matches the class of the objects. In the ideal case, all objects from class 1 are ranked higher than all objects from class 0, yielding AUC = 1, while for totally random classification, AUC = 0.5. Due to its robustness, the AUC is preferred over accuracy in automated comparisons of multiple classifiers. Nevertheless, the classification accuracy of the final classifiers is presented alongside the AUC. Model performance was also assessed using calibration metrics, including the calibration slope, calibration intercept, Brier score, and calibration-in-the-large (CITL). The models were further evaluated using calibration plots, observed-versus-predicted risk plots across risk deciles, and decision curve analysis.

### 3.5. Minimal-optimal feature selection

The final selection of the best markers for the prediction of carotid plaque (CP) was performed using the Robust Agglomerative Feature Selection (RAFS) algorithm developed by Piliszek et al. [15]. The algorithm performs hierarchical clustering of variables using a measure of dissimilarity that takes into account the difference in information about the class variable contributed by either variable. The set of variables is then represented by representatives of each cluster. The representative selection algorithm chooses the variable that has the highest mutual information with the class variable. Clustering is performed multiple times using different subsamples of the entire data set.

The final ranking of variables is then made using the aggregate results, returning the variables most often returned as representatives at each level. The method returns alternative rankings for all possible clustering levels, from 2 up to *N*, where *N* represents no clustering. RF models were then built using top-*N* cluster representatives, for *N* ranging from 2 to 15. The final selection was made for the minimal set of variables for which the models built on this set had an AUC statistically indistinguishable from the model built with all relevant variables.

### 3.6. Software

All modelling was performed in R version 4.5.2 [16], using the randomForest version 4.7.1.2 [17], Boruta version 9.0.0 [10], MDFS version 1.5.5 [12], and RAFS version 0.2.5 [18] packages.

### 3.7. Biological relevance analysis

The biological relevance of the identified biomarkers was analysed using the STRING database [19,20]. STRING is an application that displays known and predicted interactions between user-entered biomarkers in the form of a network. Interactions are displayed based on evidence derived from co-expression, genomic neighbourhood, experimental data, curated databases, co-mentioning in PubMed abstracts (text mining), and other sources.

In the first step, a network of interactions was created using all proteins identified as relevant by Boruta. STRING version 12.0 was used with a minimum required interaction score of 0.4 (medium confidence), and all available interaction sources were included. Clustering analysis of this network was performed using the Markov Clustering (MCL) algorithm [21], with the inflation parameter set to 3. The STRING database also provides a functional enrichment analysis module, based on biological processes from the Gene Ontology database and KEGG pathways, among others. Functional enrichment terms were considered significant at an FDR below 0.05 after Benjamini–Hochberg correction for multiple testing.

In our study, we analysed the biological functions of the 25 biomarkers with the highest Boruta importance scores. The STRING functional enrichment results were used as an initial guide to the biological processes and pathways represented in this protein set. The protein descriptions and annotations available through STRING links to GeneCards, KEGG, GenBank and UniProt databases were subsequently reviewed. Finally, relevant original publications were identified and reviewed in PubMed [22–27]. On the basis of this combined evidence, the proteins were manually assigned by the authors to functional groups relevant to atherogenesis. This manual functional grouping was independent of the MCL clustering of the STRING interaction network.

### 3.8. Ethics statement

The Medical University of Bialystok’s Ethics Committee in Poland granted ethical approval for this research on 29th September 2016, under the approval number R-I-002/108/2016. The study was executed in alignment with the Declaration of Helsinki. Informed consent was obtained from all subjects.

## 4. Results

**Fig 1:**
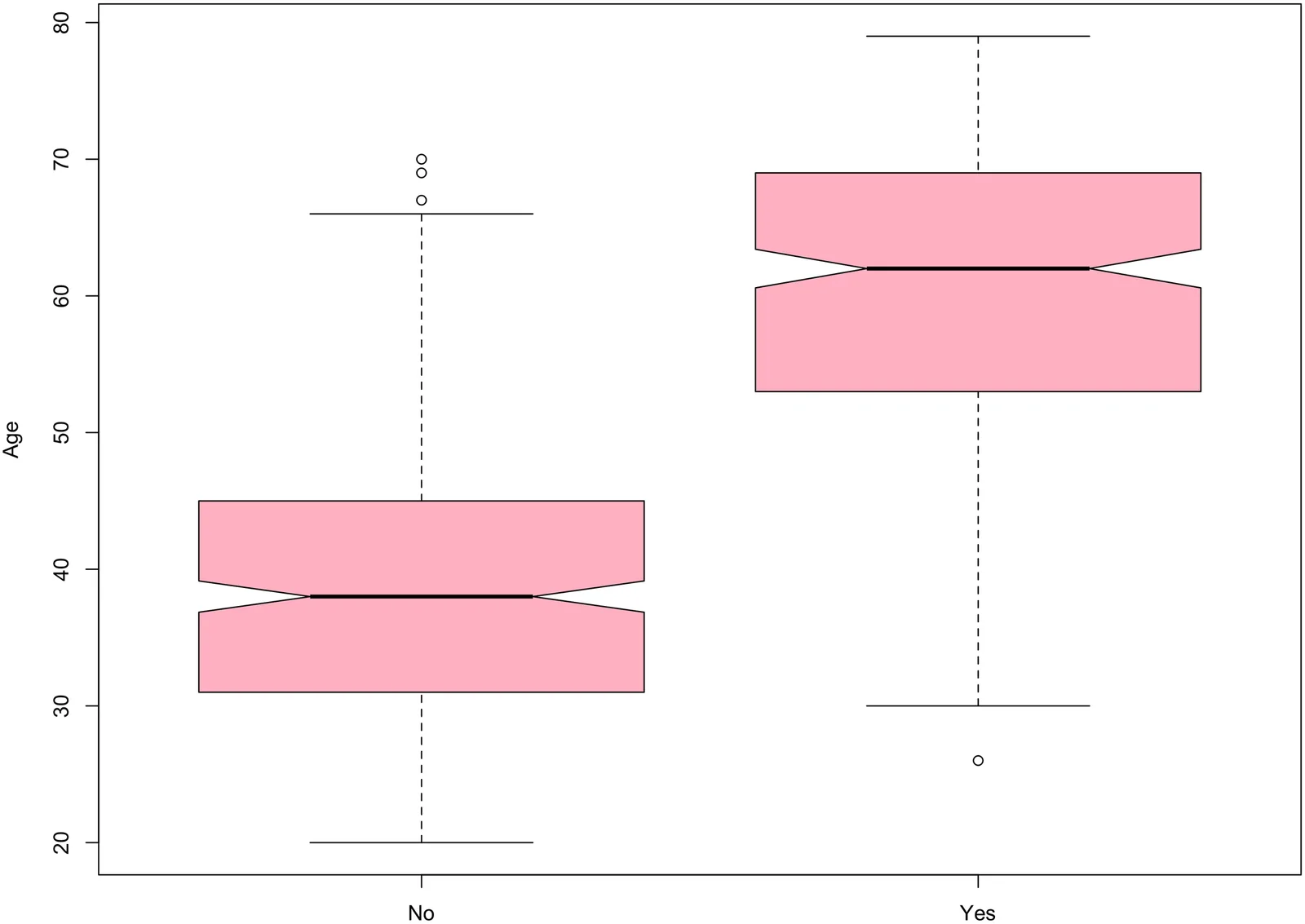
Age distribution. Age distribution in negative (left side) and positive classes (right side).

**Fig 2:**
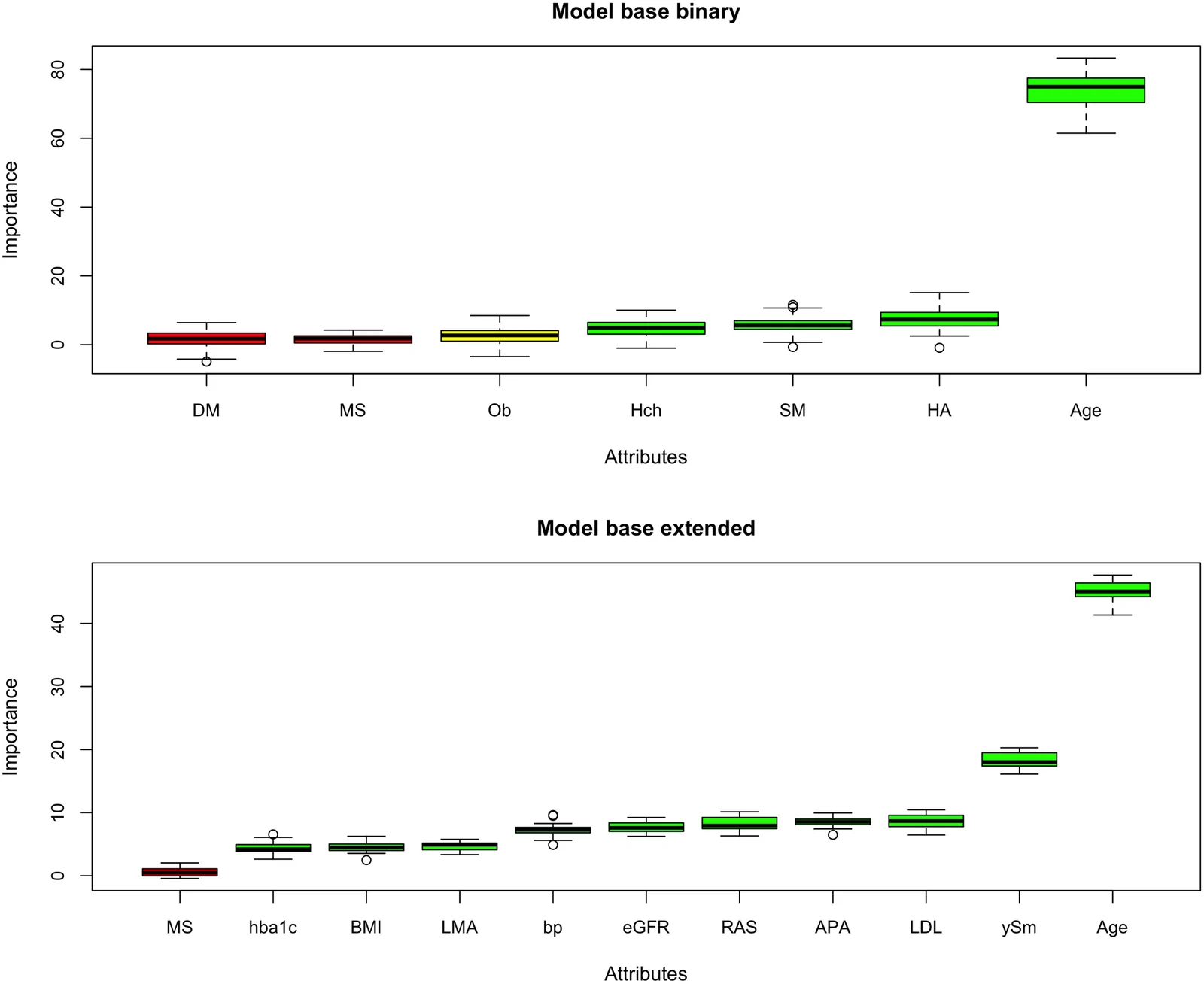
Importance from models Mbase and Mext Importance of traditional risk factors (top) (binary if possible) and extended traditional risk factors (bottom) (continuos if possible) calculated by Boruta algorithm. Green boxes – important variables; yellow boxes – tentative variables; red boxes – unimportant variables. DM – diabetes, Hch – hypercholesterolemia, HA – hypertension, BP – systolic blood pressure, Ob – obesity, Sm – smoking, MS – male sex, HbA1c - glycated hemoglobin, RAS -Renin-Angiotensin System agents, LLA – lipid-lowering agents, APA – antiplatelet agents, LDL – Low-density lipoprotein cholesterol, eGFR - Estimated glomerular filtration rate, BMI- body mass index, ySM- years of smoking.

**Fig 3:**
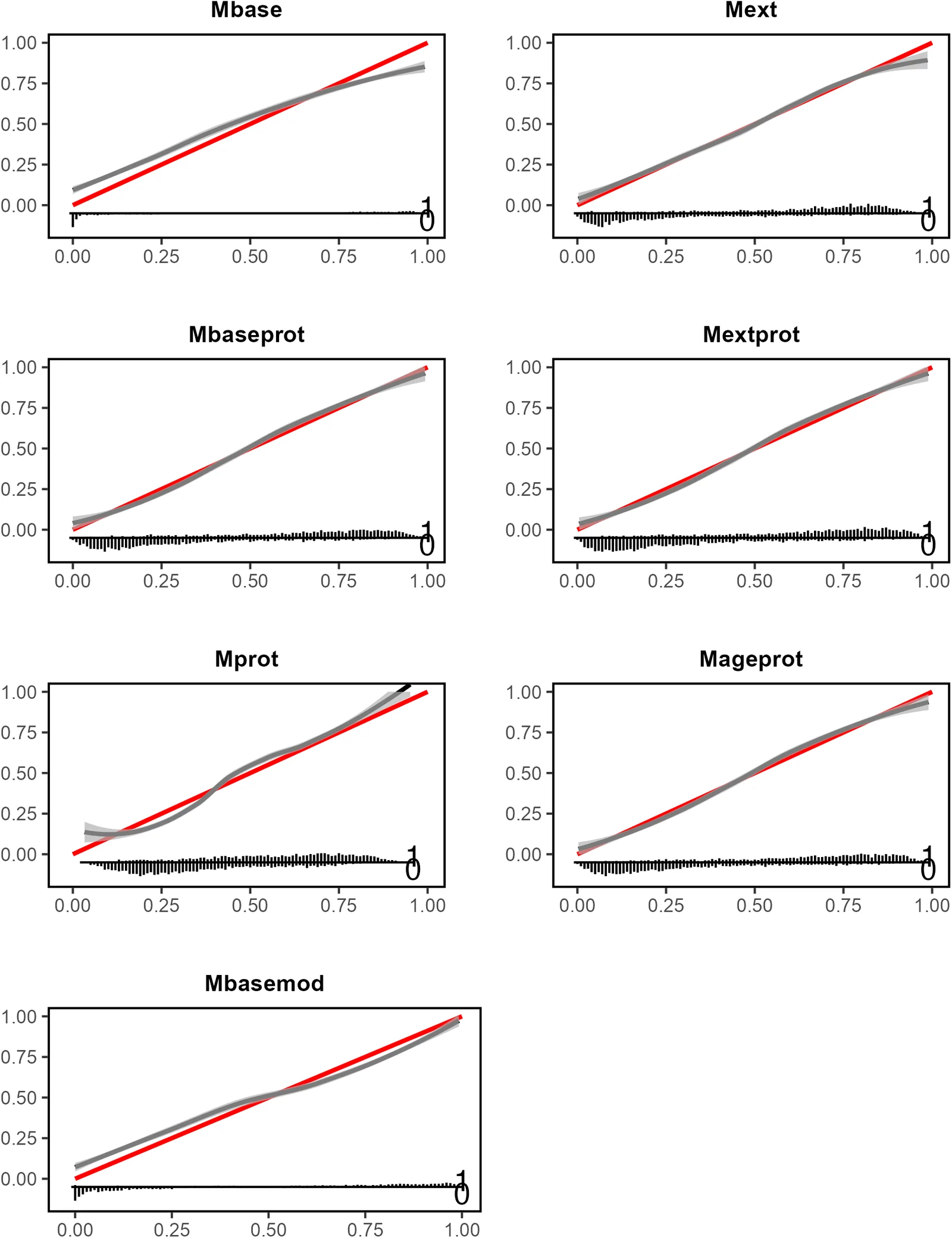
Calibration curves for all models. The red diagonal line represents the ideal calibration (where predicted risks equal observed proportions). The solid black/grey line indicates the flexible calibration curve estimated via LOESS smoothing (with the 95% confidence interval shaded in grey). The spikes along the bottom axis represent the distribution of predicted risks. Models Mbaseprot and Mextprot demonstrated the best calibration performance among all evaluated models, showing near-perfect agreement between predicted risks and observed outcomes across the entire probability spectrum.

**Fig 4:**
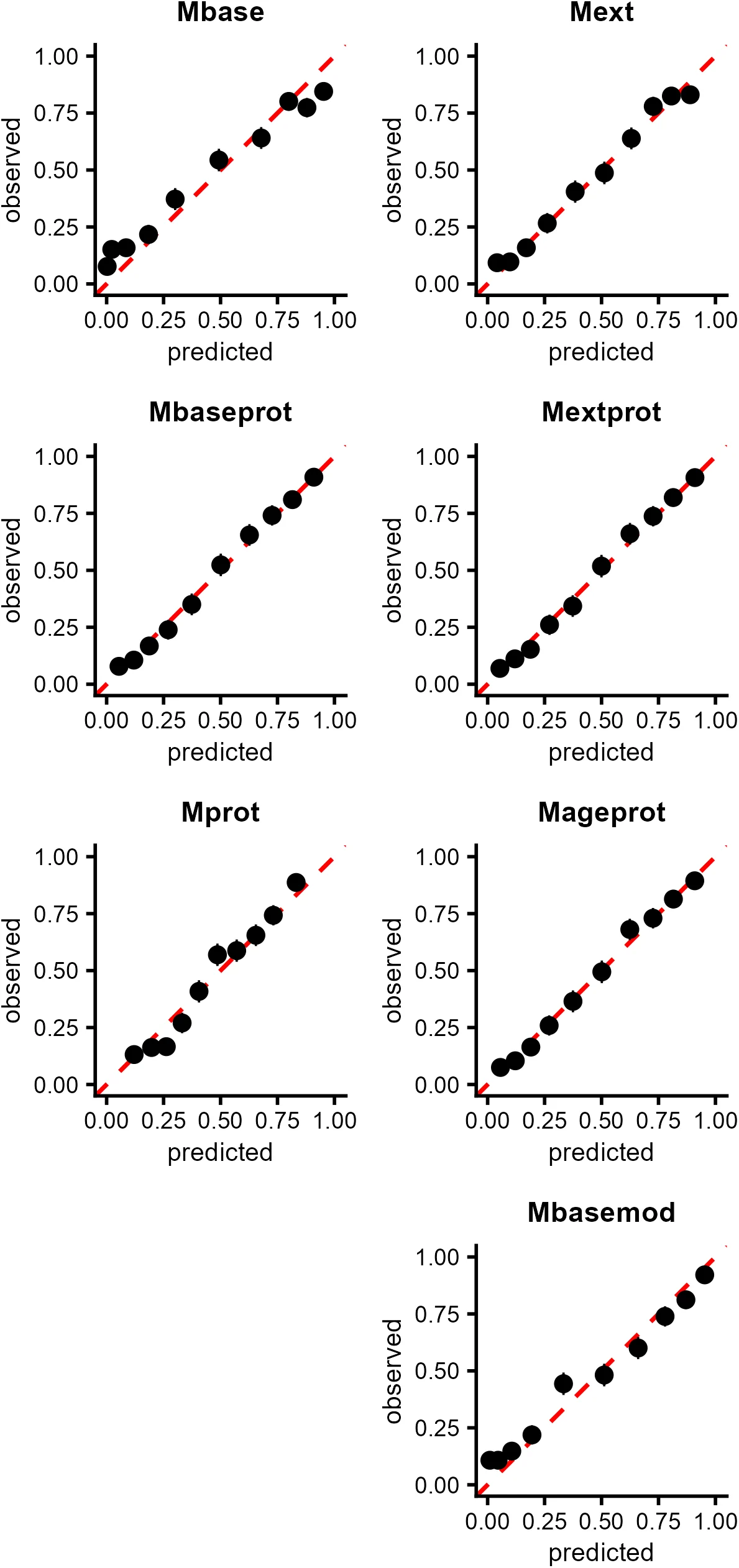
Observed-versus-predicted risk across risk deciles for all models. The red dashed line represents perfect calibration (*y* = *x*). Black dots indicate the grouped patient percentiles with corresponding confidence intervals. Models Mbaseprot, Mextprot, and Mageprot demonstrate the highest calibration accuracy, closely aligning with the ideal diagonal line across the entire risk spectrum, while minor deviations are observed in models Mbase, Mext, Mprot, and Mbasemod.

The study group comprised 698 consecutive participants. Proteomic measurements were missing for five random subjects for technical reasons. The final analysed group included 693 participants (median age 47 years, interquartile range 36–62; 43.4% were male [n = 301]). CP was detected in 320 cases (46.2%). Detailed clinical characteristics of the study group are presented in Table 1 and were already published elsewhere [3]. We noticed that in our dataset, all people under 30 years of age did not have CP, and all people over 70 years of age did; see Fig 1. Therefore, further analyses were limited to participants aged 30–70 (N = 548). In the first step, we have built the model consisting of traditional risk factors for atherosclerosis: age, male sex, diabetes, hypercholesterolemia, obesity, hypertension, and smoking. In this case, we achieved AUC = 0.825 (Table 2, model Mbase). We also performed tests of the importance of particular risk factors using the Boruta algorithm. “Age” appeared to be the most important variable, “male sex” and “diabetes” was not significant, see Fig 2. In the next step, we expanded the model by replacing binary variables with continuous ones and adding renal function and pharmacotherapy. This model was calles “Mext” and included: age, male sex, glycated hemoglobin (HbA1c), LDL cholesterol, body mass index (BMI), systolic blood pressure, years of smoking (in current smokers), estimated glomerular filtration rate (eGFR), the use of agents acting on the renin–angiotensin system, lipid-lowering drugs, and antiplatelet agents. More features were identified as important in this model, see Fig 2. Although the discriminative performance was similar for models Mbase and Mext (AUC: 0.825 vs. 0.826), model Mext exhibited markedly better calibration, as demonstrated by improved calibration metrics, calibration plots, and closer agreement between observed and predicted risk across risk deciles, see Table 2, Table 3, Fig 3 and 4.

**Table 1:** Clinical characteristics according to the presence of carotid plaques (CPs).

	Patients with CPs	Patients without CPs	p-value
	(n = 320)	CPs(n = 373)	p-value
Age (years)	62 (53–69)	38 (31–45)	<0.001
Sex, men (n,%)	145 (45.3%)	156 (41.8%)	0.356
Current smoking (n,%)	74 (23.1%)	73 (19.6%)	0.254
Hypertension (n,%)	206 (64.4%)	107 (28.6%)	<0.001
Diabetes (n,%)	70 (21.9%)	23 (6.2%)	<0.001
Hypercholesterolaemia (n,%)	273 (85.3%)	220 (60.0%)	<0.001
BP systolic (mmHg)	128.5 (116.62–140.25)	119.3 (108.5–130.5)	<0.001
BP diastolic (mmHg)	82 (76.5–88.75)	80 (73.5–86.5)	0.002
BMI (kg/m^2^)	27.8 (24.34–31.24)	25.05 (22.59–28.36)	<0.001
**Biochemistry**			
Fasting glucose (mmol/L)	5.72 (5.39–6.17)	5.39 (5.06–5.72)	<0.001
HbA1c (%)	5.6 (5.4–5.9)	5.2 (5.0–5.5)	<0.001
LDL-cholesterol (mmol/L)	3.40 (2.59–4.03)	3.05 (2.47–3.54)	<0.001
HDL-cholesterol (mmol/L)	1.52 (1.26–1.84)	1.59 (1.32–1.89)	0.184
Triglycerides (mmol/L)	1.16 (0.90–1.64)	0.94 (0.68–1.38)	<0.001
hsCRP (mg/L)	0.83 (0.41–1.65)	0.59 (0.27–1.44)	<0.001
Interleukin-6 (pg/mL)	3.07 (2.09–4.12)	2.34 (1.83–3.51)	<0.001
**Pharmacotherapy**			
Beta-blockers (n,%)	104 (32.5%)	25 (6.7%)	<0.001
ACE-inhibitors or sartans (n,%)	100 (31.3%)	34 (9.1%)	<0.001
Diuretics (n,%)	32 (10.0%)	10 (2.7%)	<0.001
Lipid-lowering drugs (n,%)	80 (25.0%)	16 (4.3%)	<0.001
Antidiabetic drugs (n,%)	34 (10.6%)	9 (2.4%)	<0.001
Antiplatelet or anticoagulant drugs (n,%)	65 (20.3%)	8 (2.1%)	<0.001

BP—blood pressure; BMI—body mass index; LDL—low-density lipoprotein; HDL—high-density lipoprotein; hsCRP—high-sensitivity C-reactive protein; ACE—angiotensin-converting enzyme.

**Table 2:** AUC calibration parameters.

Model	AUC	95% Confidence	Brier	Calibration	Calibration	CITL
		interval	score	slope	intercept	
Mbase	0.825	0.811 - 0.833	0.174	0.522	0.078	0.155
Mext	0.827	0.814 - 0.836	0.169	0.965	0.02	0.041
Mprot	0.795	0.781 - 0.805	0.185	1.21	0.017	−0.006
Mbaseprot	0.841	0.829 - 0.849	0.162	1.073	−0.009	0.003
Mextprot	0.844	0.830 - 0.852	0.161	1.093	0.001	0.001
Mageprot	0.839	0.826 - 0.847	0.163	1.077	−0.004	−0.003
Mbasemod	0.838	0.823 - 0.845	0.166	0.697	0.043	0.094

Mbase – traditional risk factors (age, male sex, diabetes, hypercholesterolemia, obesity, hypertension and smoking, binary values if possible),Mext – extended traditional risk factors (age, male sex, HbA1c, LDL cholesterol, BMI, systolic blood pressure, years of smoking, eGFR, the use of agents acting on the renin–angiotensin system, lipid-lowering drugs, and antiplatelet agents), Mprot – proteomic variables, Mbaseprot – proteomic plus traditional risk factors, Mextprot- proteomic plus Mext data, Mageprot – proteomic plus “age” and “male sex”, Mbasemod – traditional risk factors plus artificial variable based on proteins. Abbreviations: AUC – Area Under the ROC Curve, CITL - calibration-in-the-large.

**Table 3:** Models evaluation.

treshold	0.5					Youden index					Class prevalence				
Model	Acc	Prec	Se	MCC	DOR	Acc	Prec	Se	MCC	DOR	Acc	Prec	Se	MCC	DOR
Mbase	0.759	0.746	0.726	0.518	10.9	0.754	0.767	0.678	0.508	11.0	0.759	0.735	0.746	0.518	11.10
Mext	0.756	0.735	0.736	0.511	10.5	0.752	0.764	0.675	0.503	10.5	0.755	0.716	0.774	0.513	10.6
Mprot	0.723	0.711	0.672	0.443	7.4	0.712	0.713	0.642	0.426	7.2	0.729	0.696	0.731	0.459	8.2
Mbaseprot	0.767	0.751	0.739	0.533	12.1	0.755	0.791	0.641	0.511	13.0	0.771	0.740	0.775	0.543	12.8
Mextprot	0.770	0.755	0.740	0.538	12.8	0.757	0.795	0.644	0.516	13.4	0.775	0.744	0.778	0.551	13.7
Mageprot	0.769	0.752	0.742	0.534	12.6	0.753	0.789	0.644	0.508	13.6	0.766	0.735	0.770	0.533	12.4
Mbasemod	0.761	0.743	0.736	0.520	11.1	0.755	0.766	0.681	0.508	11.1	0.758	0.723	0.759	0.517	10.9

Evaluation of modelling: Mbase – traditional risk factors (age, male sex, diabetes, hypercholesterolemia, obesity, hypertension and smoking, binary values if possible),Mext – extended traditional risk factors (age, male sex, HbA1c, LDL cholesterol, BMI, systolic blood pressure, years of smoking, eGFR, the use of agents acting on the renin–angiotensin system, lipid-lowering drugs, and antiplatelet agents), Mprot – proteomic variables, Mbaseprot – proteomic plus traditional risk factors, Mextprot- proteomic plus Mext data, Mageprot – proteomic plus “age” and “male sex”, Mbasemod – traditional risk factors plus artificial variable based on proteins. Abbreviations: Acc – Accuracy, Prec – Precision, Se – Sensitivity, MCC – Matthews Correlation Coefficient, DOR – Diagnostic Odds Ratio.

The next step was to build models using only proteomic variables. In this case, the Boruta algorithm found 42 important proteins in each cross-validation fold. The most important protein factors were found to be GDF-15, CDCP1, CHIT1, Flt3L, CDH2 and CRTAC1. The 25 most important variables are reported in Table 4. List of all important proteins is in S2 Table. Table 5 shows the distribution of the most important proteins in the negative and positive classes. Among them, GDF-15 definitely stood out. Tests conducted on proteomic biomarkers showed an AUC of 0.795 (Table 2, Table 3, model Mprot).

**Table 4:** Twenty five most important proteomic variables returned by Boruta.

Protein	GDF-15	CDCP1	CHIT1	FLT3LG	CDH2	CRTAC1	TFPI	SOST	TNFRSF11B
Importance	19.8	14.0	12.2	8.7	8.5	8.1	8.1	7.7	7.2
Protein	FAS	LGALS4	PLAT	KIT	CR2	CTSD	SIGLEC7	REG4	ACP5
Importance	6.6	6.4	6.4	6.3	6.3	6.0	5.9	5.7	5.7
Protein	CXCL9	NPPB	CSTB	SERPINE1	TFF2	IGFBP7	EFEMP1	...	**sMax**
Importance	5.6	5.6	5.4	5.3	4.8	4.8	4.8	...	3.2

ShadowMax (sMax) is the most important contrast variable created by Boruta algorithm in the feature selection process.

**Table 5:** Distribution of twenty-five most important proteomic variables returned by Boruta in negative and positive classes.

Protein	GDF-15	CDCP1	CHIT1	FLT3LG	CDH2	CRTAC1	TFPI	SOST	TNFRSF11B
no CP	4.59	2.96	4.25	9.65	2.88	3.80	9.10	4.37	3.49
CP	5.09	3.41	4.83	9.88	3.18	4.04	9.26	4.65	3.68
p-value	$2.2\times 10^{-16}$	$2.2\times 10^{-16}$	$3.11\times 10^{-8}$	$1.97\times 10^{-10}$	$1.34\times 10^{-13}$	$3.22\times 10^{-8}$	$5.99\times 10^{-8}$	$4.99\times 10^{-12}$	$6.04\times 10^{-12}$
Protein	FAS	LGALS4	PLAT	KIT	CR2	CTSD	SIGLEC7	REG4	ACP5
no CP	5.88	4.01	5.14	5.03	8.02	3.40	3.37	8.41	4.89
CP	6.06	4.29	5.50	4.88	7.82	3.60	3.56	8.71	5.09
p-value	$9.14\times 10^{-8}$	$1.69\times 10^{-10}$	$1.01\times 10^{-9}$	$8.06\times 10^{-7}$	$4.7\times 10^{-6}$	$3.19\times 10^{-10}$	$1.44\times 10^{-10}$	$6.45\times 10^{-10}$	$4.43\times 10^{-6}$
Protein	CXCL9	NPPB	CSTB	SERPINE1	TFF2	IGFBP7	EFEMP1	...	...
No CP	6.84	2.73	4.45	8.23	4.96	8.62	5.50	...	...
CP	7.19	3.14	4.71	8.17	5.25	8.80	5.65	...	...
p-value	$4.72\times 10^{-9}$	$8.54\times 10^{-8}$	$3.19\times 10^{-10}$	0.04	$2.44\times 10^{-6}$	$6.42\times 10^{-8}$	$4.43\times 10^{-6}$	...	...

We have extended the proteomic model to include age and male sex as well. It was far more influential than the others. The model proved to be better than the one based on protein variables only. Its AUC was 0.839 (Tables 2 and 3, model Mageprot).

We continued to expand the feature set. We added all the traditional risk factors to the proteomic variables. The resulting models were not significantly different from those outlined above (Tables 2 and 3, model Mbaseprot). The next model (Mextprot) was developed by combining proteomic data with an extended set of traditional clinical risk factors from Mext model. In this case, both the AUC and the calibration parameters were slightly better.

In an additional model, we combined traditional risk factors and an artificial variable derived from predicting the presence of plaques using proteomic variables. In this case, we obtained AUCs equal to 0.838, (see Table 2 and 3, model Mbasemod).

Additional performance metrics, including accuracy, precision, Matthews correlation coefficient (MCC), and diagnostic odds ratio (DOR), were also evaluated for all models. The Mbaseprot and Mextprot models consistently achieved the best performance according to these metrics, see Table 2 and 3.

**Fig 5:**
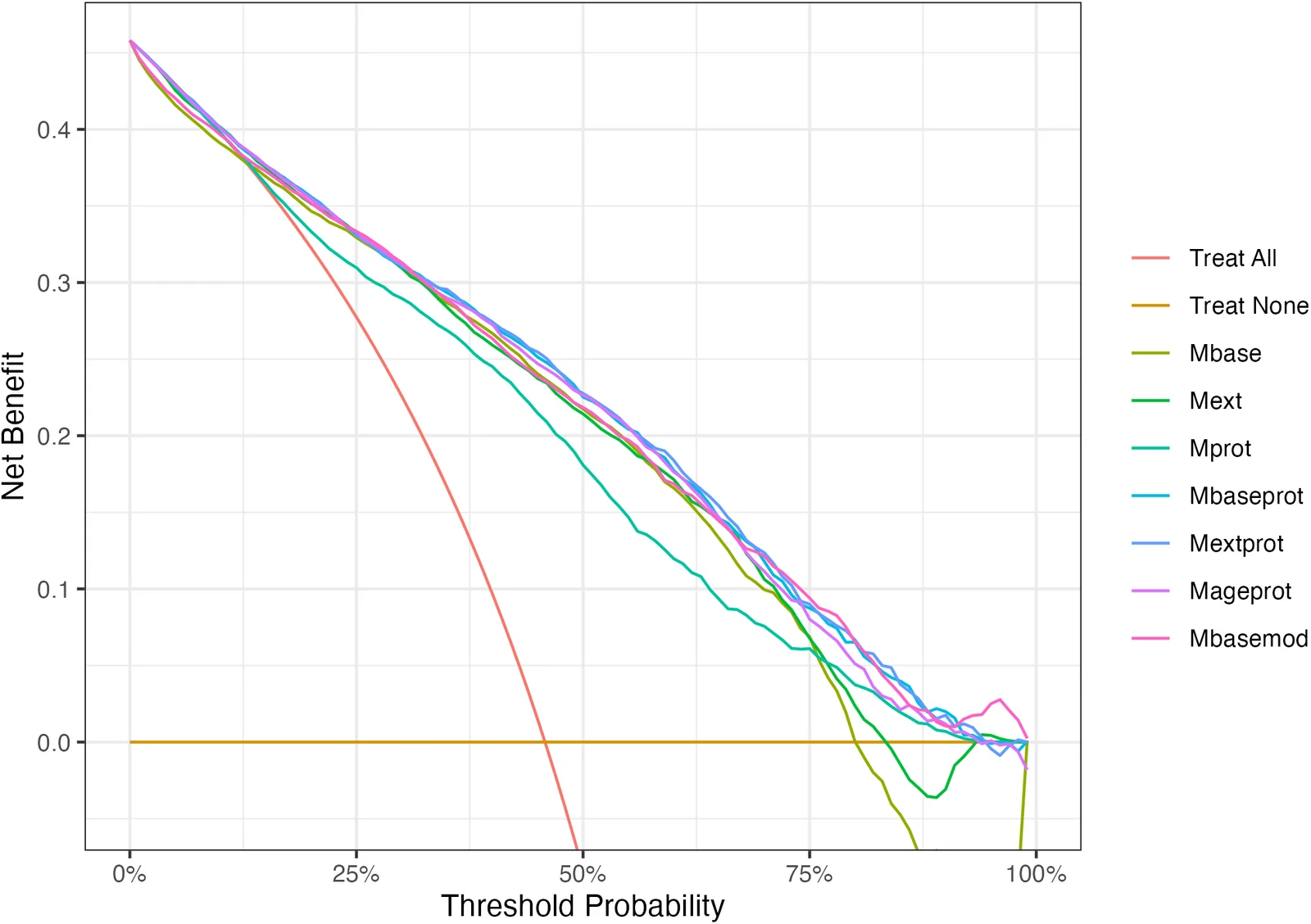
Decision-curve analysis for all models. Decision-curve analysis showed that all models yielded a positive net benefit compared with the “treat all” and “treat none” strategies across most threshold probabilities. The decision curves largely overlapped, suggesting that the models provided similar clinical utility. Although models Mbaseprot, Mextprot, and Mbasemod showed a slightly higher net benefit at some higher threshold probabilities, these differences were generally small. Mbase model appears to be the weakest, particularly in high-risk areas.

**Fig 6:**
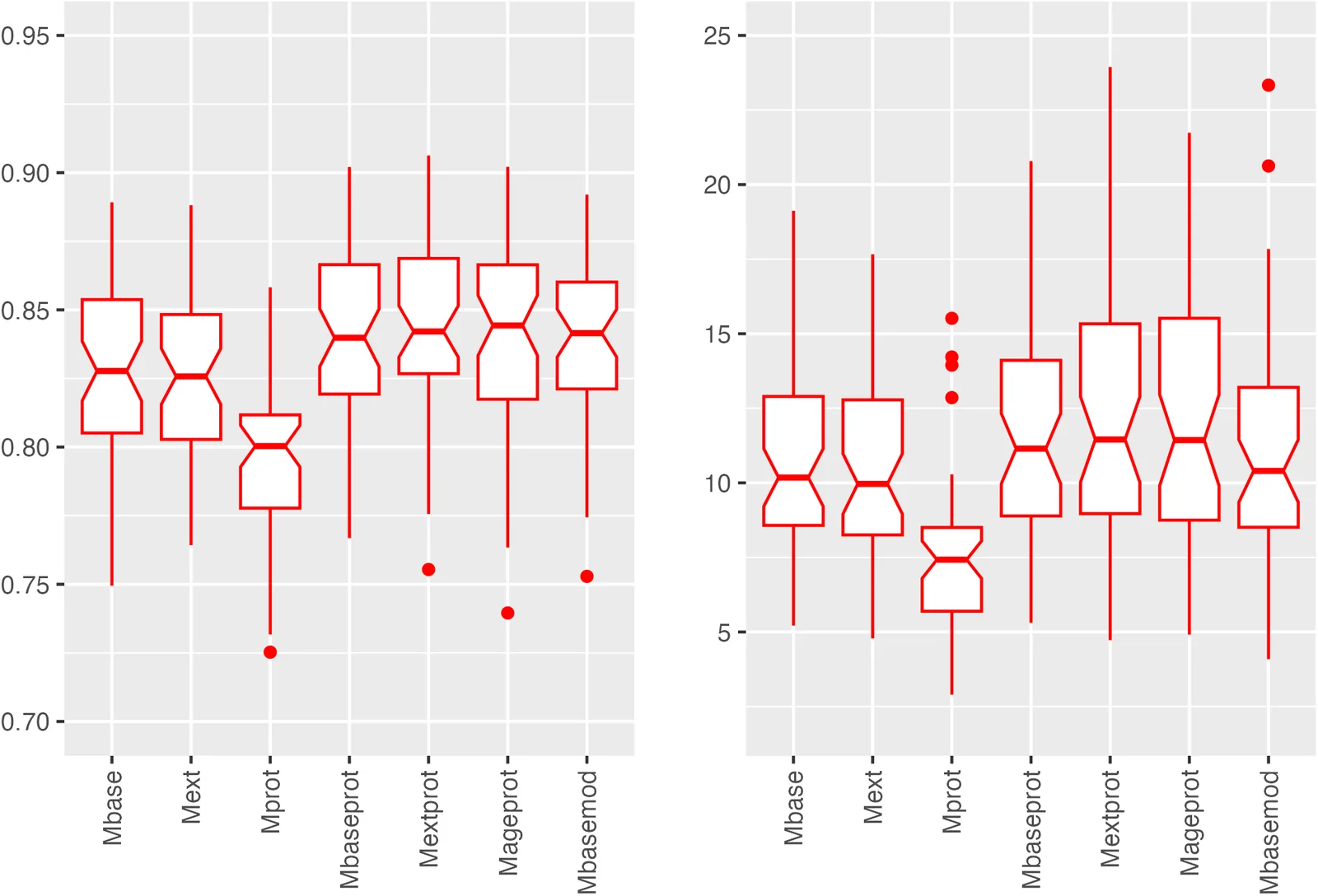
AUC and DOR distribution for all models. Distribution of AUC (left panel) and Diagnostic Odd Ratio (right panel) from 10 repetition of 5-fold cross-validation: Mbase – traditional risk factors (age, male sex, diabetes, hypercholesterolemia, obesity, hypertension and smoking, binary values if possible),Mext – extended traditional risk factors (age, male sex, HbA1c, LDL cholesterol, BMI, systolic blood pressure, years of smoking, eGFR, the use of agents acting on the renin–angiotensin system, lipid-lowering drugs, and antiplatelet agents), Mprot – proteomic variables, Mbaseprot – proteomic plus traditional risk factors, Mextprot- proteomic plus Mext data, Mageprot – proteomic plus “age” and “male sex”, Mbasemod – traditional risk factors plus artificial variable based on proteins.

To facilitate the comparison of all models, calibration plots (Fig 3), observed-versus-predicted risk plots across risk deciles (Fig 4), and decision curves (Fig 5) were generated. These plots indicate a modest advantage of models Mbaseprot and Mextprto. In contrast, model Mbase appears to perform less favorably than the other models. AUC and DOR distribution for all models are shown on Fig 6

We also searched for the minimum optimal feature set using the RAFS algorithm. For this purpose, we used proteomic data plus traditional risk factors. In a further approach to the RAFS procedure, we used proteomic data and ‘age’. In this case, we obtained AUC = 0.830 for data set consist of 7 variables. ALL RAFS results are in Table 6.

**Table 6:** Searching for minimal optimal feature set using RAFS. First 9 steps.

RAFS steps - “traditional risk factors” + proteins									
Feature set	age, GDF-15	+ CDCP1	+CHIT1	+TNFRSF11B	+Hypertension	+CDH2	+SOST	+FLT3LG	+CRTAC1
AUC	0.782	0.808	0.812	0.818	0.820	0.816	0.823	0.827	0.830
RAFS steps - “age” + proteins									
Feature set	age, GDF-15	+ CDCP1	+CHIT1	+TNFRSF11B	+CDH2	+SOST	+FLT3LG	+CSTB	+CRTAC1
AUC	0.782	0.808	0.812	0.818	0.816	0.823	0.830	0.830	0.827

Traditional risk factors: age, male sex, diabetes, hypercholesterolemia, obesity, hypertension and smoking.

To investigate the associations between proteins and age, two types of analyses were performed. First, age was predicted from proteomic data in cross-validation loop. The resulting model achieved a Pearson correlation coefficient squared (*R*^2^) of 0.663 and a root mean squared error (RMSE) equal to 7.14. Feature selection revealed that many of the proteins selected for plaque prediction also contributed to age prediction (e.g., GDF-15, CDCP1, SOST, CXCL9, FLt3L). Next, separate proteomics-based models for atherosclerosis were developed for four age groups (30–39, 40–49, 50–59, and 60–70 years). For the 30–39-year age group, the model achieved an AUC of 0.630 (95% CI: 0.605–0.655). For the 40–49-year group, the AUC was 0.600 (95% CI: 0.579–0.621). The corresponding AUCs were 0.608 (95% CI: 0.583–0.632) for the 50–59-year group and 0.607 (95% CI: 0.578–0.636) for the 60–70-year group.There was a moderate correlation between age and proteome. Despite this, the predictive values of the models were comparable in all age groups (the AUC was lower than in the whole study group, probably due to smaller sample sizes).

**Fig 7:**
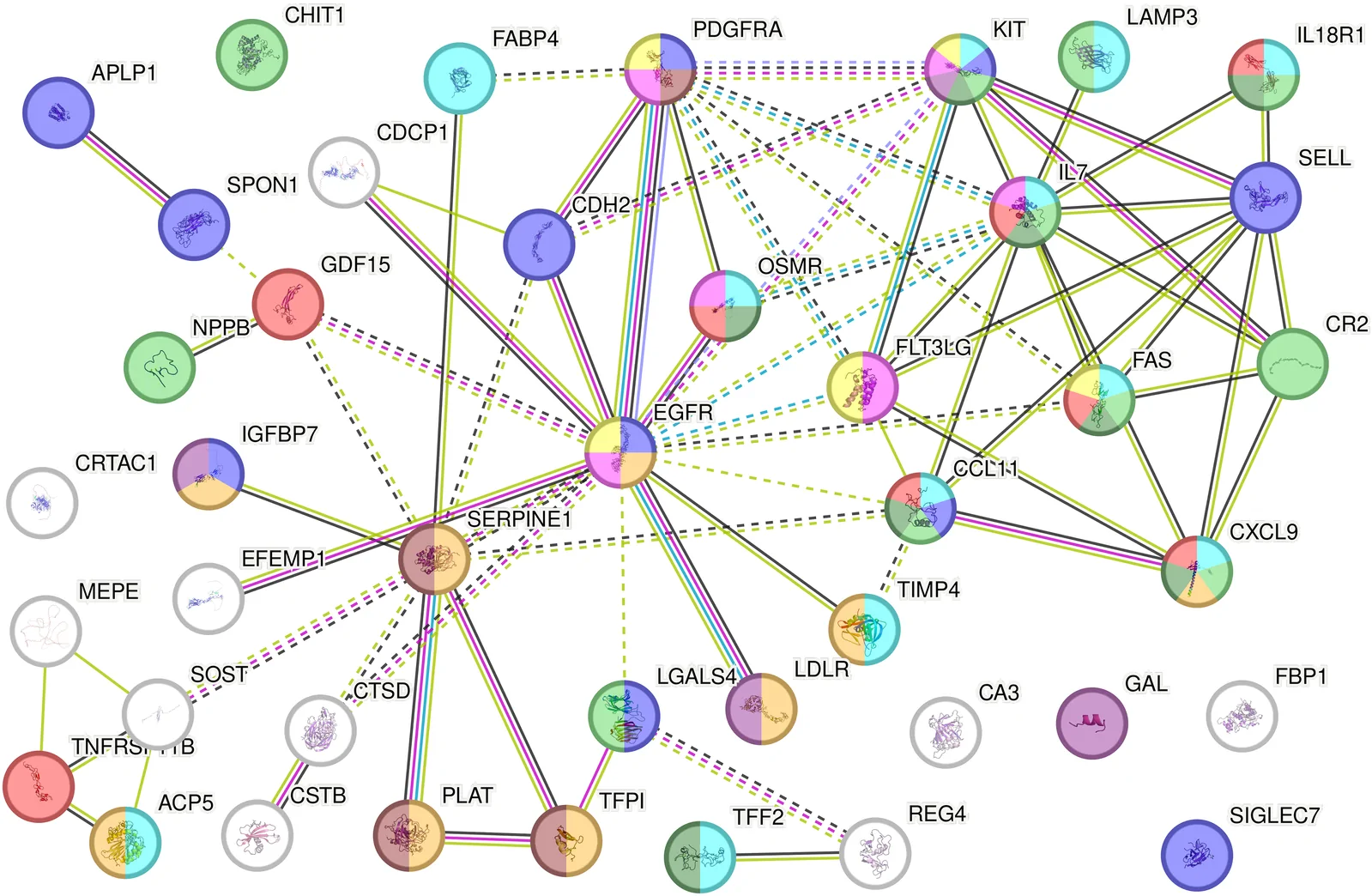
Graph depicting fourty-two marker proteins relevant for the age-restricted dataset. Graph was generated with the help of the web interface of the STRING database (version 12.0). The edges of the graph correspond to the interactions between proteins identified in the database. Colours correspond to the gene ontology and KEGG pathways that are significantly enriched within the set of these proteins and that are relevant to the molecular processes underlying carotid atherosclerosis. GO terms: GO:0034097 Response to cytokine (cyan), GO:0007155 Cell adhesion (lavender blue), GO:0006955 Immune response (lime green), GO:0033993 Response to lipid (peach yellow), GO:0019221 Cytokine-mediated signalling pathway (shadow green), GO:0030195 Negative regulation of blood coagulation (light mauve), GO:0019218 Regulation of steroid metabolic process (purple). KEGG pathways: hsa04060 Cytokine-cytokine receptor interaction (light salmon pink), hsa04151 PI3K-Akt signaling pathway (blush pink) and hsa04010 MAPK signaling pathway (yellow). Permanent link: https://version-12-0.string-db.org/cgi/network?networkId=bQZnso4pYIPo.

All the proteins that were significantly associated with CP based on the Boruta algorithm were subjected to analysis in the STRING database, and we built a network of potential interactions between all these biomarkers, see Fig 7. We obtained a very complex model in which only 6 proteins showed no interactions. The other 36 proteins formed the nodes of the network with 72 edges (potential interactions) between them (average node degree 3.44). The expected number of edges in a random set of 42 proteins is 21, and p-value for the graph is less then 10^–16^. The biomarker with the highest importance value in the Boruta analysis was GDF-15, which showed interaction with 4 biomarkers, including EGFR, SERPINE1, NT-proBNP [NPPB] and SPON 1. Most of these potential interactions were based on text mining or gene co-expression scoring, in the case of the EGFR also on putative homologs interacting in experimental data in organisms other than humans. Based on Gene Ontology database (included in functional enrichment in your network function of the STRING database), GDF-15 was involved in cytokine-cytokine receptor interaction together with FAS (tumor necrosis factor receptor superfamily member 6), TNFRSF11B, IL18R1, IL7, CCL11, CXCL9 and OSMR

Very limited information is available for the CDCP1 protein in the STRING database. It has only two statistically significant links with other proteins in our network- CDH2 and EGFR. In the case of EGFR, there is evidence of coexpression, direct interaction and also joint appearance in the abstract of the publication. Neither CHIT1 nor CRTAC1 was described to interact with any of the other biomarkers studied.

The CDH2 is an adhesion molecule linked with CDCP1, SERPINE1 (by text mining and gene co-expression), PDGFRA, EGFR, and KIT. The latter three associations are also based on experimental data, and the molecules are significantly associated with the cell adhesion process in the functional enrichment module. The other biomarkers that were involved in cell adhesion but did not show association with CDH2 were: SELL, CCL11, LGALS4, SPON1, APLP1, IGFBP7 and SIGLEC7. FLT3LG interacts with several biomarkers: EGFR, CCL11, KIT, SELL, PDGFRA, IL7 and CXCL9. Again, most of the interactions result from text mining or gene co-expression. In addition, FLT3LG is involved in signalling pathways: Ras (with KIT, EGFR, PDGFRA), Pl3K-Akt (with KIT, EGFR, PDGFRA, IL7 and OSMR, no direct interaction between FLT3LG and OSMR was found); MAPK (with KIT, EGFR, PDGFRA, and FAS; there is no direct interaction with FAS). FLT3LG with IL-7, CXCL9 and CCL11 were involved in cytokine activity (together with GDF-15 and TNFRSF11B that did not interact with FLT3LG).

**Fig 8:**
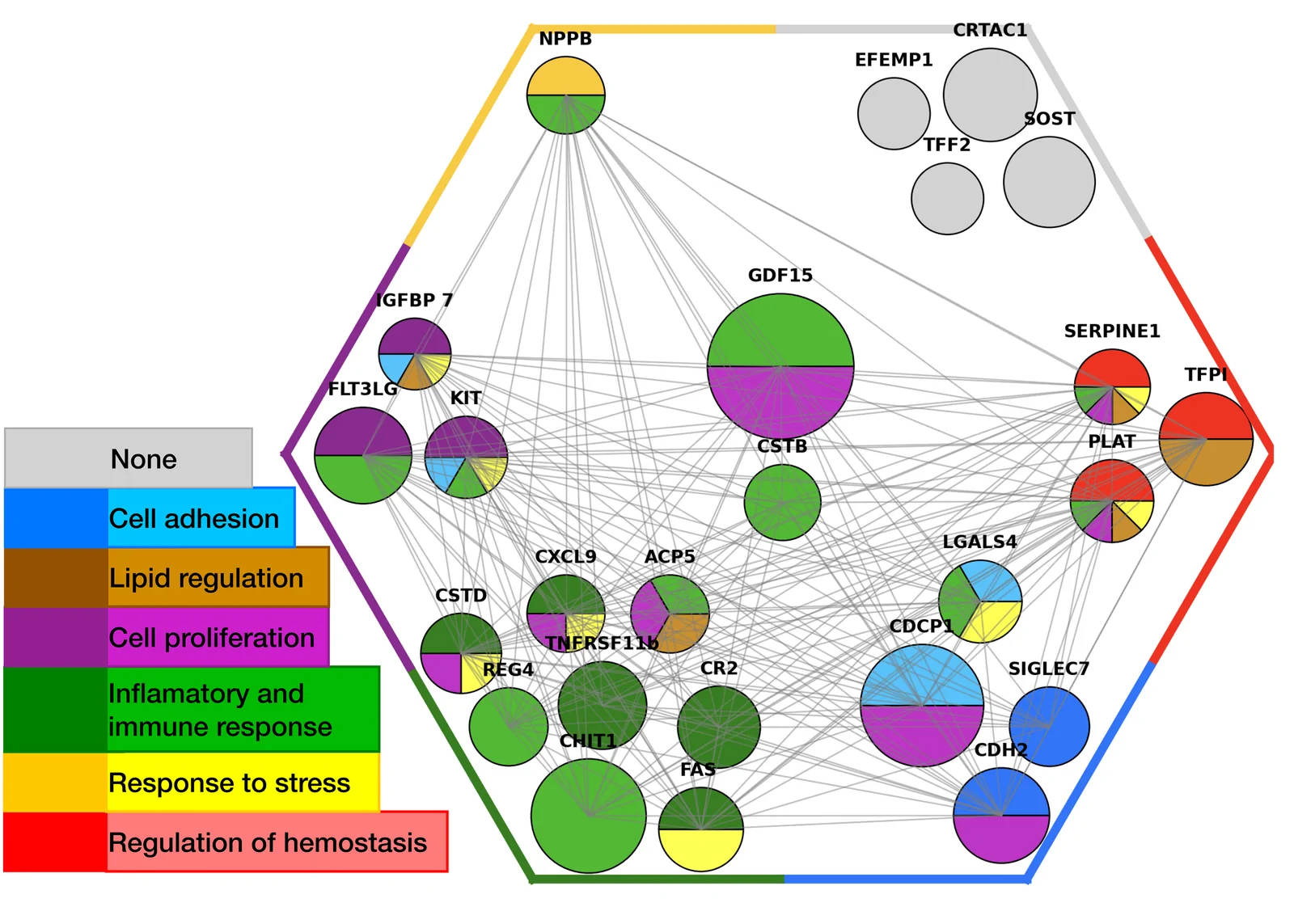
Functional grouping of the 25 most relevant markers. The node area is proportional to the Boruta importance score. Coloured sectors indicate functional classes manually assigned by the authors, with the darker sector denoting the primary class. The six vertices of the hexagonal layout correspond to the six categories occurring as primary assignments, including one category for proteins not assigned to any functional class. Lipid regulation occurred only as a secondary assignment and therefore has no separate vertex. Nodes are positioned according to their primary class, and edges connect proteins sharing at least one assigned functional class.

Based on manual clustering of the function of the 25 biomarkers with the highest importance scores, four clusters of biological processes potentially associated with atherogenesis were identified (Fig 8). The largest cluster was related to the inflammatory and immune response (stained green). They were the predominant function of 5 biomarkers (CR2, CSTD, CXCL9, TNFRSF11b, FAS). Further clusters involved processes related to cell adhesion (CDH2, SIGLEC7, cluster stained blue), proliferation (KIT, FLT3LG, IGFBP 7; stained purple) and haemostasis (TFPI, PLAT, SERPINE1; stained red). We could not reliably define the dominant effect for some of the proteins, either because they were multifunctional with no major function or because their function was poorly described and speculative. The cellular proliferation cluster contained the association with Pl3K-Akt or MAPK/ERK relay pathways. Response to stress was the primary function of one biomarker – NT-proBNP (NPPB; function stained yellow). Some of the proteins were also involved in lipid regulation (stained brown), but in neither case was this a major function. Four biomarkers could not be associated with any of the above functions (SOST, CRTAC1, TFF2, EFEMP). For CSTB, data on a possible function related to atherogenesis were insufficient.

## 5. Discussion

CP is a common condition in the general population, associated primarily with age. In our study, we estimated the risk of CP only in participants aged 30−70 years, since there were no cases of CP in those aged < 30 years, and all those aged > 70 years had CP. A cohort of 548 participants was analysed by machine learning using clinical data and laboratory data of 460 proteomic biomarkers related to cell metabolism, cell adhesion, and immune or inflammatory response. Fourty two biomarkers were associated with CP. However, in all the models we created, the most important proteins, in a decreasing order were: GDF-15, CDCP1, CHIT1, FLT3LG, CDH2 and CRTAC1. All models had good performance as measured by AUC, and all statistical parameters describing them. The proteomic model (Mprot, based on proteomic variables alone, without clinical context) was almost as good as the traditional risk factor model (Mbase). The joint models (Mextprot, Mageprot, Mbaseprot) had the best performance. However, when the other risk factors were added to the proteomic variables combined with age and sex, the model’s performance did not improve further. The RAFS analysis showed that age was the most important clinical variable in algorithm creation. These analyses show that proteomic data imprinted on a drop of blood have good internal cross-validated discrimination for predicting CP, with performance that in our dataset is comparable to that of a traditional clinical model.

To the best of our knowledge, there are currently no clinical risk scales in use for predicting the presence of CPs. In our study, we obtained a multivariate proteomic signature of this clinical condition. The risk scores used in practice relate to the 10-year risk of fatal and non-fatal cardiovascular disease events (SCORE2) [28] or the clinical likelihood of obstructive atherosclerotic coronary artery disease [29]. Risk scores such as the Carotid Artery Risk Score or Carotid Plaque Score are used to assess the risk of stroke in patients with CPs or CS [30,31].

The importance value for age was several times higher than for all the other risk factors. Binary risk factors showed a lower importance score. The main advantage of standard risk assessment based on conventional risk factors is considered to be simplicity. However, in clinical practice, data are often either incomplete or unreliable. When assessing the risk of atherosclerosis, the diagnosis of hypertension, hypercholesterolemia, or diabetes is initially based on the patient’s medical history. This should be supplemented by specific tests. However, even this approach does not consider the level of compensation of these diseases (blood pressure levels, LDL cholesterol, or HBA1C concentrations), which are key parameters. In addition, the predictive value of current smoking or smoking history is limited, depending on the number of pack-years smoked or when smoking was stopped. A common approach is to consider all these variables as qualitative, whereas the abnormalities can be more accurately described by appropriate continuous parameters. Proteomic model, on the other hand, is completely objective, and its availability may increase dramatically in the future. In our study, the model incorporating risk factors as continuous variables did not demonstrate better predictive value than the model using binary variables. However, the calibration measures were markedly better. Due to the limitations of our study design, we cannot draw any causal conclusions; we can only infer the utility of the panels as a CP fingerprint.

Importantly, our aim was not to identify proteins associated with CP independently of age and conventional risk factors, but to identify a multivariate proteomic signature. Because age was by far the strongest predictor of plaque status in our analyses, its relationship with the proteomic signature requires particular consideration. Age is associated with numerous biological processes, and many age-related proteomic changes may therefore correlate with plaque status simply because both vary with age. Still, at least some of these proteomic signals can be expected to reflect biological processes associated with plaque formation beyond their shared relationship with chronological age. Since we cannot identify these processes and the related proteins a priori, we deliberately seek a broad multivariate signature rather than a small set of independently associated markers.

For this reason, we selected the 25 proteins with the highest Boruta importance scores rather than restricting the analysis to the minimal-optimal RAFS set. This pragmatic cutoff retained a broader panel and reduced dependence on the particular correlated proteins selected in a given sample, especially because substantially fewer variables were confirmed in the smaller cross-validation training sets.

The selected proteins are therefore interpreted jointly as a hypothesis-generating signature, not as independently associated or causally implicated biomarkers. This approach also improves robustness. Random Forest combines information from multiple correlated variables and averages their predictive signal, reducing the impact of biological and measurement variability in individual proteins.

The proteins with the highest importance scores include GDF-15, CDCP1, CHIT1, FLTG3L, CDH2, CRTAC1, and TFPI. They are not among the main proteins involved in the pathophysiological pathways leading to atherosclerosis. In all cases, the nature of their association with atherosclerosis remains unclear. We do not have data on a cause-and-effect relationship, so they may only be biomarkers of this process. Interestingly, 2 of these biomarkers (CHIT1 and CRTAC1), despite having a very high importance score, were not associated with the other biomarkers studied in the STRING analysis, indicating that they may be part of pathways not yet described in the pathophysiology of atherosclerosis.

Of the biomarkers we evaluated, GDF-15 (growth differentiation factor 15) had the highest importance score. GDF-15 is expressed in a wide range of cell types [32], with increased biomarker levels observed in response to tissue hypoxia, acute injury, and oxidative stress. GDF-15 is involved in the regulation of apoptosis, angiogenesis, cell repair, and cell growth [24]. In the heart, it protects cardiomyocytes against ischemia-reperfusion injury [33]. Increased concenttations of GDF-15 are associated with lower risk adverse events in patients with NSTEMI [34,35], heart failure [36] or stable CAD [37]. It corresponded with vascular function (pulse wave velocity and flow-mediated dilation) in the Framingham Heart Study [38]. GDF-15 level has also been associated with intima-media thickness in Swedish cohorts [39,40]. The researchers showed that the rs1054564 single nucleotide polymorphism in GDF-15 is associated with the occurrence of carotid plaques [41]. In this study, we confirm that this biomarker is increased in participants with CP. The mechanism od this association, however, remains unclear.

GDF-15 is a member of the transforming growth factor-beta (TGF-beta) superfamily. This protein binds to several TGF-beta receptors, leading to the recruitment and activation of SMAD family transcription factors. GDF-15 leads to activation of the ERK pathway, based on studies on brain injury in mice [42]. It promotes proliferation, migration, and tube formation of endothelial cells in vitro through the activation of Src and its downstream AKT, MAPK, and NF-κB signaling, which was proved on a colony of mice hepatocellular carcinoma (HCC) cells [24]. Tobacco smoke impairs the Akt kinase/endothelial nitric oxide synthase (Akt/eNOS) pathway and endothelial wound healing, which, together with an increase in GDF-15 induced by the presence of oxidative stress, Nrtf2, OSGIN1/2 and turbulent flow in arteries, impairs the body’s anti-atherosclerotic defences [43]. Chinese studies have shown that GDF-15 through binding to GDNF family receptor alpha-like (GFRAL) and RET can dose-independently inhibit ADP-induced platelet aggregation. GDF-15 can also inhibit ADP-induced protein kinase B (AKT) and ERK activation in platelets [25].

The second most important protein in our main model is CUB domain-containing protein 1 (CDCP1). It plays a crucial role in cancer cell survival and growth, metastasis, and resistance to treatment. It is associated with, among others, signaling cascades SFK-CDCP1-PKCδ, PI3K/AKT, and RAS/ERK [44]. CDCP1 concentrations were increased in patients with myocardial infarction [45], with an additional increase in cases of ventricular fibrillation [46]. A Mendelian randomization study identified CDCP1 as novel risk factors for coronary artery disease [47]. In a genome-wide analysis, copy number variations in the CDCP1 region (3p21.31) were associated with hyperlipidemia and myocardial infarction [48]. In another study, CDCP1 plasma levels correlated with cognitive impairment [49]. Its role in carotid atherosclerosis remains to be elucidated, but CA may be a link between CDCP1 and cognitive function.

Chitinase 1 (CHIT1) is mainly secreted by activated macrophages and epithelial cells. There is a clear association between CHIT1 expression and lipid-laden macrophages within atherosclerotic vessel walls [50]. Interestingly, based on an experimental study in mice, CHIT1 has a protective effect against atherosclerosis by inhibiting inflammatory responses converting macrophages to an M2 phenotype, and promoting lipid uptake and cholesterol efflux in macrophages [51]. CHIT1 also interacts with TGF-β1 to augment fibroblast TGF-β receptors 1 and 2 expression and TGF-β–induced Smad and MAPK/ERK activation [52]. Polymorphisms in CHIT1 are associated with the prevalence of calcified plaques in the coronary arteries [53]. CHIT1 levels are increased in former smokers or tend to be a male-specific biomarker for incident heart failure [54,55]. Interestingly, despite evidence of an association with atherosclerotic processes, CHIT1 did not show an association with any other biomarker we examined.

FMS-like tyrosine kinase 3 ligand (FLT3LG) is a member of the FLT3 family of cytokines and is involved in infection recognition and activation of immune responses through a feedback loop between dendritic cells (DCs) and regulatory T cells [26]. Research in mice shows that a significant number of FLT3LG-dependent dendritic cells are found in the tunica intima of the artery containing the atherosclerotic plaque, where they play an antiatherosclerotic role [26]. In analyses of atherosclerotic plaques, the presence of FLT3LG was associated with abnormally regulated macrophages. The presence of this protein was associated with increased immune infiltration and high instability of atherosclerotic plaques [56]. This protein has also been shown to be associated with ischemic heart disease [57].

Cadherins are adhesion molecules that are also found on atherosclerotic plaque cells. Cadherin-2 (CDH2) also known as cadherin N, mediates interaction between endothelial cells and pericytes. Hyperlipidemia leads to reduced CDH2 expression on microvascular cells in the mouse brain, resulting in reduced pericyte coverage and microvascular dysfunction [58]. CDH2 is overexpressed in aortic vascular smooth muscle cells (VSMC) from patients with myocardial infarction undergoing coronary artery bypass grafting [59]. CDH2-mediated intercellular contacts reduce VSMC apoptosis, whereas its cleavage enhances this process and may therefore increase plaque development and rupture [60]. CDH2 shedding promoted by MMP-12 or MMP-9 leads to increased VSMC proliferation [27].

Of particular note is cartilage acidic protein 1 (CRTAC1), which we show is not currently associated with any of the other proteins we studied, but, in our material, is strongly associated with carotid plaques. CRTAC1 was first identified as a marker that distinguishes chondrocytes from osteoblasts and mesenchymal stem cells. Since then, its increased expression has been shown in diseases such as osteoarthritis [61], acute ischemic stroke [62] or bladder cancer [63]. However, the only paper describing its association with atherosclerosis is a single-cell meta-analysis describing a comprehensive genetic study of scRNA-seq of atherosclerotic plaque cells in coronary artery disease. It identified high scRNA expression of CRTAC1 in arterial smooth muscle cells and concluded that it could be used as a marker of plaque stability and a promising indicator of atherosclerosis progression [64]. The exact pathways by which CRTAC1 interacts with other proteins in the pathogenesis of carotid atherosclerosis are still unknown and require further study.

Based on manual clustering of the functions of 25 key biomarkers, 4 clusters of biological processes potentially associated with atherogenesis were identified. Most of the 25 proteins included in this analysis were associated with the inflammatory and/or immune response. This was followed by cell adhesion and proliferation, which is consistent with the current concept of atherogenesis. Some of the proteins studied were markers of haemostasis. Interestingly, no biological function potentially associated with atherogenesis was found for four biomarkers (SOST, CRTAC1, TFF2, EFEMP1). On the other hand, several biomarkers were involved in many processes simultaneously, as exemplified by KIT, SERPINE1 or CXCL9.

The cell proliferation cluster contained the PI3K-Akt or MAPK/ERK signalling pathways. These pathways are related and have been implicated in atherogenesis [65]. Phosphatidylinositol 3-kinase (PI3K) is a signalling molecule involved in the regulation of many physiological and pathophysiological processes. The main downstream effector is AKT (protein kinase B). PI3K-AKT signalling is implicated in smooth muscle proliferation, but also in adhesion molecule expression, endothelial cell apoptosis, macrophage autophagy or phenotypic transformation [65]. The p38 mitogen-activated protein kinase (MAPK) is linked to the extracellular signal-regulated kinase (ERK), they form a common MAPK/ERK pathway that is responsible for gene regulation of processes including cell proliferation, differentiation, senescence, stress response, migration and apoptosis [66]. This pathway is critical for microvascular endothelial cell survival and apoptosis [67]. The influence of the p38/MAPK pathway on the progression of atherosclerotic changes has been observed, but the underlying mechanisms are very complex and not fully described [66].

There are already studies on proteomics in atherosclerosis. They focus either on the composition of atherosclerotic plaques [68,69] or the serum/plasma proteome [70]. Most analyses of the proteomic composition of atherosclerosis tissues demonstrate the key influence of inflammation-related immune cells (mainly neutrophils and macrophages) through the presence of proteins specifically associated with them [71]. In a study using a “foam cell” model based on its stimulation with oxLDL, 59 proteins were identified whose levels were significantly increased and 17 proteins whose levels were significantly decreased. With regard to our results, similarity was found only in the presence of the FABP4 protein [68].

In a research analysing plaques from carotid arteries obtained by endarterectomy, overexpression of 95 proteins and downregulation of 117 proteins was observed [72]. The SAPHIR study identified 4 matrix biomarkers associated with adverse outcomes in CA (matrix metalloproteinase 9, S100A8/S100A9, cathepsin D and galectin-3-binding protein) [73].

Plasma proteome studies are technically easier to perform than plaque composition studies, and the results are potentially easier to apply in practice. The prospective Swedish PRIVUS study examined the levels of 92 cardiovascular disease-related biomarkers (OLINK panel) in the plasma of seniors. They found 7 proteins that were significantly associated with the occurrence of carotid plaques. These proteins are TNFRSF11B, HAVCR1/TIM1, GDF-15, MMP-12, REN, TNFSF14 and GH1. Of these, GDF-15 and TNFRSF11B had also high importance in our study [74].

The ML methods we used represent a different approach to traditional statistical methods, which focus mainly on statistical significance, the exclusion of null hypotheses and, in the case of associations, the independence of other factors. As a result, true observations may be excluded, especially regarding rare conditions. ML, on the other hand, focuses on the importance of observed associations that may have direct practical implications. The Boruta machine learning algorithm has been used to analyse proteomic data in cardiovascular disease. It enabled the identification of 18 biomarkers from the OLINK inflammatory and cardiovascular panel that were associated with an increased risk of CV death in patients with chronic coronary syndrome. These proteins were classified according to their involvement in the following pathomechanisms: myocardial strain- dysfunction-hypertrophy-fibrosis, myocyte death and apoptosis, renal injury, haemodynamic stress, RAS activation, vascular inflammation and immune modulation [70]. In other studies, the algorithm helped describe plasma signatures indicating high platelet reactivity to aspirin and clopidogrel [75] and identified five biomarkers that were independently associated with myocardial infarction after adjustment for cardiovascular risk factors (CDCP1, CD6, IL18-R1, IL-6 and CXCL1). Their twofold plasma concentrations were correlated with a threefold increased risk of MI [45]. In comparison, in our study, CDCP1 showed the association with CPs. In 5893 patients with atrial fibrillation from the ARISTOTLE and RE-LY cohorts, 9 out of 268 biomarkers associated with cardiovascular death were identified [76]. In different analyses of this cohort, a further 9 biomarkers were found to be associated with an increased risk of bleeding, including GDF-15 as the second most important biomarker [77].

### 5.1. Limitations and future research directions

In this study, we only focused on the presence of CPs as a marker of cardiovascular risk. We did not study the morphology of CPs, which is a highly specialised procedure that should be performed by experienced physicians, not technicians. High-risk CP morphology has a strong documented association with stroke risk, regardless of the degree of stenosis. However, an incorrect interpretation of atherosclerotic plaque morphology by unqualified personnel could lead to legal issues. The other parameters, like plaque burden, number and location of plaques, were not analysed, which limits clinical and mechanistic interpretation of our findings. The next limitation of our study is the cross-sectional design. The biomarkers we have identified as being associated with CPs should be validated in other populations, and their causative role in atherogenesis should be tested in prospective analyses. Given the aforementioned limitations, the study does not permit the identification of cause-and-effect relationships.

## 6. Conclusion

In our study we developed an ML-based fingerprint of CP which showed good internal cross-validated discrimination in subjects aged 30–70 years and comprised 25 biomarkers. Its diagnostic odds ratio was comparable to a traditional clinical model (12.1 vs. 11.6). We identified 42 significant biomarkers associated with CP. The six most important proteins were: GDF-15, CDCP1, CHIT1, FLT3LG, CDH2 and CRTAC1. Among the biomarkers, GDF-15 had definitely the highest importance score. The potential association of the top biomarkers with atherosclerosis is highly speculative and only hypothesis-generating. Based on manual clustering of the functions of 25 key biomarkers, 4 clusters of biological processes potentially associated with atherogenesis were identified. Most of the 25 proteins included in this analysis were associated with the inflammatory and immune response. This was followed by cell adhesion and proliferation, consistent with the current concept of atherogenesis. Our findings require validation in prospective studies, as well as in different cohorts with different genetic backgrounds, lifestyles, and environments. They do not permit the identification of cause-and-effect relationships. Nevertheless, they are worth considering for further mechanistic studies.

## Supporting information

S1 Table List of biomarkers. Abbervation names from OLINK database, STRING – Abbervation names from STRING database.
(PDF)

S2 Table All important proteins in age-restricted group. (PDF)
