# Supplementary material for: Proteomic machine-learning signatures associated with carotid plaque: A population-based hypothesis-generating study
Source: PLoS One. 2026 Sep 24;21(9):e0358765. doi: 10.1371/journal.pone.0358765 (PMC13606957; doi:10.1371/journal.pone.0358765)
Supplement: Supplementary file 1 [file pone.0358765.s001.pdf]

S1 Table: List of biomarkers

No.	OLINK	STRING	Full name
1	4E-BP1	EIF4BP1	Eukaryotic translation initiation factor 4E-binding protein 1
2	ACP6	ACP6	Lysophosphatidic acid phosphatase type 6
3	ADA	ADA	Adenosine Deaminase
4	ADGRE2	ADGRE2	Adhesion G protein-coupled receptor E2
5	ADGRG2	ADGRG2	Adhesion G-protein coupled receptor G2
6	AHCY	AHCY	Adenosylhomocysteinase
7	ALCAM	ALCAM	CD166 antigen
8	ALDH1A1	ALDH1A1	Retinal dehydrogenase 1
9	ANG	ANG	Angiogenin
10	ANGPT2	ANGPT2	Angiopoietin-2
11	ANGPTL1	ANGPTL1	Angiopoietin-related protein 1
12	ANGPTL3	ANGPTL3	Angiopoietin-related protein 3
13	ANGPTL7	ANGPTL7	Angiopoietin-related protein 7
14	ANXA11	ANXA11	Annexin A11
15	ANXA4	ANXA4	Annexin A4
16	AOC3	AOC3	Membrane primary amine oxidase
17	AP-N	ANPEP	Aminopeptidase N
18	APEX1	APEX1	DNA-(apurinic or apyrimidinic site) lyase
19	APLP1	APLP1	Amyloid-like protein 1
20	APOM	APOM	Apolipoprotein M
21	AREG	AREG	Amphiregulin
22	ARG1	ARG1	Arginase-1
23	ARNT	ARNT	Aryl hydrocarbon receptor nuclear translocator
24	ARTN	ARTN	Artemin
25	AXIN1	AXIN1	Axin-1
26	AXL	AXL	Tyrosine-protein kinase receptor UFO
27	AZU1	AZU1	Azurocidin
28	BACH1	BACH1	Transcription regulator protein BACH1
29	BAG6	BAG6	Large proline-rich protein BAG6
30	Beta-NGF	NGF	Beta-nerve growth factor
31	BIRC2	BIRC2	Baculoviral IAP repeat-containing protein 2
32	BLM hydrolase	BLMH	Bleomycin hydrolase
33	BTN3A2	BTN3A2	Butyrophilin subfamily 3 member A2
34	C1QTNF1	C1QTNF1	Complement C1q tumor necrosis factor-related protein 1
35	C2	C2	Complement C2
36	CA1	CA1	Carbonic anhydrase 1
37	CA13	CA13	Carbonic anhydrase 13
38	CA3	CA3	Carbonic anhydrase 3
39	CA4	CA4	Carbonic anhydrase 4

No.	OLINK	STRING	Full name
40	CANT1	CANT1	Soluble calcium-activated nucleotidase 1
41	CASP-3	CASP3	Caspase-3
42	CASP-8	CASP8	Caspase-8
43	CCDC80	CCDC80	Coiled-coil domain-containing protein 80
44	CCL11	CCL11	Eotaxin
45	CCL14	CCL14	C-C motif chemokine 14
46	CCL15	CCL15	C-C motif chemokine 15
47	CCL16	CCL16	C-C motif chemokine 16
48	CCL18	CCL18	C-C motif chemokine 18
49	CCL19	CCL19	C-C motif chemokine 19
50	CCL20	CCL20	C-C motif chemokine 20
51	CCL23	CCL23	C-C motif chemokine 23
52	CCL24	CCL24	C-C motif chemokine 24
53	CCL25	CCL25	C-C motif chemokine 25
54	CCL28	CCL28	C-C motif chemokine 28
55	CCL3	CCL3	C-C motif chemokine 3
56	CCL4	CCL4	C-C motif chemokine 4
57	CCL5	CCL5	C-C motif chemokine 5
58	CD163	CD163	Scavenger receptor cysteine-rich type 1 protein M130
59	CD164	CD164	Sialomucin core protein 24
60	CD1C	CD1C	T-cell surface glycoprotein CD1c
61	CD244	CD244	Natural killer cell receptor 2B4
62	CD28	CD28	T-cell-specific surface glycoprotein CD28
63	CD2AP	CD2AP	CD2-associated protein
64	CD40	CD40	CD40L receptor
65	CD46	CD46	Membrane cofactor protein
66	CD5	CD5	T-cell surface glycoprotein CD5
67	CD59	CD59	CD59 glycoprotein
68	CD6	CD6	T cell surface glycoprotein CD6 isoform
69	CD79B	CD79B	B-cell antigen receptor complex-associated protein beta chain
70	CD83	CD83	CD83 antigen
71	CD8A	CD8A	T-cell surface glycoprotein CD8 alpha chain
72	CD93	CD93	Complement component C1q receptor
73	CDCP1	CDCP1	CUB domain-containing protein 1
74	CDH1	CDH1	Cadherin-1
75	CDH2	CDH2	Cadherin-2
76	CDH5	CDH5	Cadherin-5
77	CDHR5	CDHR5	Cadherin-related family member 5
78	CDSN	CDSN	Corneodesmosin
79	CES1	CES1	Liver carboxylesterase 1
80	CFHR5	CFHR5	Complement factor H-related protein 5
81	CHI3L1	CHI3L1	Chitinase-3-like protein 1

No.	OLINK	STRING	Full name
82	CHIT1	CHIT1	Chitotriosidase-1
83	CHL1	CHL1	Neural cell adhesion molecule L1-like protein
84	CHRDL2	CHRDL2	Chordin-like protein 2
85	CKAP4	CKAP4	Cytoskeleton-associated protein 4
86	CLEC4A	CLEC4A	C-type lectin domain family 4 member A
87	CLEC4C	CLEC4C	C-type lectin domain family 4 member C
88	CLEC4D	CLEC4D	C-type lectin domain family 4 member D
89	CLEC4G	CLEC4G	C-type lectin domain family 4 member G
90	CLEC5A	CLEC5A	C-type lectin domain family 5 member A
91	CLEC6A	CLEC6A	C-type lectin domain family 6 member A
92	CLEC7A	CLEC7A	C-type lectin domain family 7 member A
93	CLMP	CLMP	CXADR-like membrane protein
94	CLSTN2	CLSTN2	Calsyntenin-2
95	CLUL1	CLUL1	Clusterin-like protein 1
96	CNDP1	CNDP1	Beta-Ala-His dipeptidase
97	CNTN1	CNTN1	Contactin-1
98	CNTNAP2	CNTNAP2	Contactin-associated protein-like 2
99	COL18A1	COL18A1	Collagen alpha-1(XVIII) chain
100	COL1A1	COL1A1	Collagen alpha-1(I) chain
101	COMP	COMP	Cartilage oligomeric matrix protein
102	COMT	COMT	Catechol O-methyltransferase
103	CPA1	CPA1	Carboxypeptidase A1
104	CPB1	CPB1	Carboxypeptidase B
105	CR2	CR2	Complement receptor type 2
106	CRKL	CRKL	Crk-like protein
107	CRTAC1	CRTAC1	Cartilage acidic protein 1
108	CSF-1	CSF1	Macrophage colony-stimulating factor 1
109	CST3	CST3	Cystatin-C
110	CST5	CST5	Cystatin D
111	CSTB	CSTB	Cystatin-B
112	CSTD	CTSD	Cathepsin D
113	CTSH	CTSH	Pro-cathepsin H
114	CTSO	CTSO	Cathepsin O
115	CTSZ	CTSZ	Cathepsin Z
116	CX3CL1	CX3CL1	Fractalkine
117	CXADR	CXADR	Coxsackievirus and adenovirus receptor
118	CXCL1	CXCL1	C-X-C motif chemokine 1
119	CXCL10	CXCL10	C-X-C motif chemokine 10
120	CXCL11	CXCL11	C-X-C motif chemokine 11
121	CXCL12	CXCL12	Stromal cell-derived factor 1
122	CXCL16	CXCL16	C-X-C motif chemokine 16
123	CXCL5	CXCL5	C-X-C motif chemokine 5
124	CXCL6	CXCL6	C-X-C motif chemokine 6

No.	OLINK	STRING	Full name
125	CXCL9	CXCL9	C-X-C motif chemokine 9
126	DAB2	DAB2	Disabled homolog 2
127	DAPP1	DAPP1	Dual adapter for phosphotyrosine and 3-phosphotyrosine and 3-phosphoinositide
128	DCBLD2	DCBLD2	Discoidin, CUB and LCCL domain-containing protein 2
129	DCTN1	DCTN1	Dynactin subunit 1
130	DDC	DDC	Aromatic-L-amino-acid decarboxylase
131	DDX58	DDX58	Probable ATP-dependent RNA helicase DDX58
132	DEFA1_DEFA1B	DEFA1 / DEFA1B	Defensin 1 and defensin 2
133	DFFA	DFFA	DNA fragmentation factor subunit alpha
134	DGKZ	DGKZ	Diacylglycerol kinase zeta
135	DIABLO	DIABLO	Diablo homolog, mitochondrial
136	DLK-1	DLK1	Protein delta homolog 1
137	DNER	DNER	Delta and Notch-like epidermal growth factor-related receptor
138	DPP10	DPP10	Inactive dipeptidyl peptidase 10
139	DPP4	DPP4	Dipeptidyl peptidase 4
140	DPP7	DPP7	Dipeptidyl peptidase 2
141	EDAR	EDAR	Tumor necrosis factor receptor superfamily member EDAR
142	EFEMP1	EFEMP1	EGF-containing fibulin-like extracellular matrix protein 1
143	EGFR	EGFR	Epidermal growth factor receptor
144	EGLN1	EGLN1	Egl nine homolog 1
145	EIF4G1	EIF4G1	Eukaryotic translation initiation factor 4 gamma 1
146	EIF5A	EIF5A	Eukaryotic translation initiation factor 5A-1
147	EN-RAGE	S100A12	Protein S100-A12
148	ENG	ENG	Endoglin
149	ENO2	ENO2	Gamma-enolase
150	ENPP7	ENPP7	Ectonucleotide pyrophosphatase/phosphodiesterase family member 7
151	ENTPD5	ENTPD5	Ectonucleoside triphosphate diphosphohydrolase 5
152	Ep-CAM	EPCAM	Epithelial cell adhesion molecule
153	EPHB4	EPHB4	Ephrin type-B receptor 4
154	F11	F11	Coagulation factor XI
155	F7	F7	Coagulation factor VII
156	FABP4	FABP4	Fatty acid-binding protein, adipocyte
157	FAM3B	FAM3B	Protein FAM3B
158	FAM3C	FAM3C	Protein FAM3C
159	FAP	FAP	Prolyl endopeptidase FAP
160	FAS	FAS	Tumor necrosis factor receptor superfamily member 6

No.	OLINK	STRING	Full name
161	FBP1	FBP1	Fructose-1,6-bisphosphatase 1
162	FCGR2A	FCGR2A	Low affinity immunoglobulin gamma Fc region receptor II-a
163	FCGR3B	FCGR3B	Low affinity immunoglobulin gamma Fc region receptor III-B
164	FCN2	FCN2	Ficolin-2
165	FCRL1	FCRL1	Fc receptor-like protein 1
166	FCRL3	FCRL3	Fc receptor-like protein 3
167	FCRL6	FCRL6	Fc receptor-like protein 6
168	FETUB	FETUB	Fetuin-B
169	FGF-19	FGF19	Fibroblast growth factor 19
170	FGF-21	FGF21	Fibroblast growth factor 21
171	FGF-23	FGF23	Fibroblast growth factor 23
172	FGF-5	FGF5	Fibroblast growth factor 5
173	FGF2	FGF2	Fibroblast growth factor 2
174	FKBP4	FKBP4	Peptidyl-prolyl cis-trans isomerase FKBP4
175	Flt3L	FLT3LG	Fms-related tyrosine kinase 3 ligand
176	FXYD5	FXYD5	FXYD domain-containing ion transport regulator 5
177	GAL	GAL	Galanin peptides
178	Gal-3	LGALS3	Galectin-3
179	Gal-4	LGALS4	Galectin-4
180	GALNT3	GALNT3	Polypeptide N-acetylgalactosaminyltransferase 3
181	GAS6	GAS6	Growth arrest-specific protein 6
182	GDF-15	GDF15	Growth/differentiation factor 15
183	GDNF	GDNF	Glial cell line-derived neurotrophic factor
184	GHRL	GHRL	Appetite-regulating hormone
185	GLB1	GLB1	Beta-galactosidase
186	GLRX	GLRX	Glutaredoxin-1
187	GNLY	GNLY	Granulysin
188	GP1BA	GP1BA	Platelet glycoprotein Ib alpha chain
189	GP6	GP6	Platelet glycoprotein VI
190	GRAP2	GRAP2	GRB2-related adapter protein 2
191	GRN	GRN	Granulins
192	HCLS1	HCLS1	Hematopoietic lineage cell-specific protein
193	HDGF	HDGF	Hepatoma-derived growth factor
194	HEXIM1	HEXIM1	Protein HEXIM1
195	HGF	HGF	Hepatocyte growth factor
196	HNMT	HNMT	Histamine N-methyltransferase
197	HSD11B1	HSD11B1	Corticosteroid 11-beta-dehydrogenase isozyme 1
198	ICA1	ICA1	Islet cell autoantigen 1
199	ICAM-2	ICAM2	Intercellular adhesion molecule 2
200	ICAM1	ICAM1	Intercellular adhesion molecule 1
201	ICAM3	ICAM3	Intercellular adhesion molecule 3

No.	OLINK	STRING	Full name
202	IFN-gamma	IFNG	Interferon gamma
203	IFNLR1	IFNLR1	Interferon lambda receptor 1
204	IGFBP-1	IGFBP1	Insulin-like growth factor-binding protein 1
205	IGFBP-2	IGFBP2	Insulin-like growth factor-binding protein 2
206	IGFBP-7	IGFBP7	Insulin-like growth factor-binding protein 7
207	IGFBP3	IGFBP3	Insulin-like growth factor-binding protein 3
208	IGFBP6	IGFBP6	Insulin-like growth factor-binding protein 6
209	IGFBPL1	IGFBPL1	Insulin-like growth factor-binding protein-like 1
210	IGLC2	Iglc2	Immunoglobulin lambda constant 2
211	IL-1 alpha	IL1A	Interleukin-1 alpha
212	IL-10RA	IL10RA	Interleukin-10 receptor subunit alpha
213	IL-10RB	IL10RB	Interleukin-10 receptor subunit beta
214	IL-12B	IL12B	Interleukin-12 subunit beta
215	IL-13	IL13	Interleukin-13
216	IL-15RA	IL15RA	Interleukin-15 receptor subunit alpha
217	IL-17A	IL17A	Interleukin-17A
218	IL-17C	IL17C	Interleukin-17C
219	IL-17RA	IL17RA	Interleukin-17 receptor A
220	IL-18	IL18	Interleukin-18
221	IL-18BP	IL18BP	Interleukin-18-binding protein
222	IL-18R1	IL18R1	Interleukin-18 receptor 1
223	IL-1RT1	IL1RL1	Interleukin-1 receptor type 1
224	IL-1RT2	IL1RL2	Interleukin-1 receptor type 2
225	IL-2	IL2	Interleukin-2
226	IL-20	IL20	Interleukin-20
227	IL-20RA	IL20RA	Interleukin-20 receptor subunit alpha
228	IL-22 RA1	IL22RA1	Interleukin-22 receptor subunit alpha-1
229	IL-24	IL24	Interleukin-24
230	IL-2RB	IL2RB	Interleukin-2 receptor subunit beta
231	IL-33	IL33	Interleukin-33
232	IL-4	IL4	Interleukin-4
233	IL-6RA	IL6R	Interleukin-6 receptor subunit alpha
234	IL-7	IL7	Interleukin-7
235	IL-8	IL8	Interleukin-8
236	IL10	IL10	Interleukin-10
237	IL12RB1	IL12RB1	Interleukin-12 receptor subunit beta-1
238	IL2-RA	IL2RA	Interleukin-2 receptor subunit alpha
239	IL5	IL5	Interleukin-5
240	IL6	IL6	Interleukin-6
241	IL7R	IL7R	Interleukin-7 receptor subunit alpha
242	IRAK1	IRAK1	Interleukin-1 receptor-associated kinase 1
243	IRAK4	IRAK4	Interleukin-1 receptor-associated kinase 4
244	IRF9	IRF9	Interferon regulatory factor 9

No.	OLINK	STRING	Full name
245	ITGA11	ITGA11	Integrin alpha-11
246	ITGA6	ITGA6	Integrin alpha-6
247	ITGAM	ITGAM	Integrin alpha-M
248	ITGB2	ITGB2	Integrin beta-2
249	ITGB6	ITGB6	Integrin beta-6
250	ITGB7	ITGB7	Integrin beta-7
251	ITM2A	ITM2A	Integral membrane protein 2A
252	JAM-A	F11R	Junctional adhesion molecule A
253	JUN	JUN	Transcription factor AP-1
254	KIT	KIT	Mast/stem cell growth factor receptor Kit
255	KLK10	KLK10	Kallikrein-10
256	KLK6	KLK6	Kallikrein-6
257	KLRD1	KLRD1	Natural killer cells antigen CD94
258	KPNA1	KPNA1	Importin subunit alpha-5
259	KRT19	KRT19	Keratin, type I cytoskeletal 19
260	KYAT1	KYAT1	Kynurenine-oxoglutarate transaminase 1
261	LAG3	LAG3	Lymphocyte activation gene 3 protein
262	LAMP3	LAMP3	Lysosome-associated membrane glycoprotein 3
263	LAP TGF-beta-1	TGFB1	Latency-associated peptide transforming growth factor beta-1
264	LCN2	LCN2	Neutrophil gelatinase-associated lipocalin
265	LDL receptor	LDLR	Low-density lipoprotein receptor
266	LIF	LIF	Leukemia inhibitory factor
267	LIF-R	LIFR	Leukemia inhibitory factor receptor
268	LILRA5	LILRA5	Leukocyte immunoglobulin-like receptor subfamily A member 5
269	LILRB1	LILRB1	Leukocyte immunoglobulin-like receptor subfamily B member 1
270	LILRB2	LILRB2	Leukocyte immunoglobulin-like receptor subfamily B member 2
271	LILRB4	LILRB4	Leukocyte immunoglobulin-like receptor subfamily B member 4
272	LILRB5	LILRB5	Leukocyte immunoglobulin-like receptor subfamily B member 5
273	LRIG1	LRIG1	Leucine-rich repeats and immunoglobulin-like domains protein 1
274	LRP11	LRP11	Low-density lipoprotein receptor-related protein 11
275	LTBP2	LTBP2	Latent-transforming growth factor beta-binding protein 2
276	LTBR	LTBR	Lymphotoxin-beta receptor
277	LY75	LY75	Lymphocyte antigen 75
278	LYVE1	LYVE1	Lymphatic vessel endothelial hyaluronic acid receptor 1

No.	OLINK	STRING	Full name
279	MASP1	MASP1	Mannan-binding lectin serine protease 1
280	MB	MB	Myoglobin
281	MBL2	MBL2	Mannose-binding protein C
282	MCFD2	MCFD2	Multiple coagulation factor deficiency protein 2
283	MCP-1	CCL2	Monocyte chemotactic protein 1
284	MCP-2	CCL8	Monocyte chemotactic protein 2
285	MCP-3	CCL7	Monocyte chemotactic protein 3
286	MCP-4	CCL13	Monocyte chemotactic protein 4
287	MEGF9	MEGF9	Multiple epidermal growth factor-like domains protein 9
288	MEP1B	MEP1B	Meprin A subunit beta
289	MEPE	MEPE	Matrix extracellular phosphoglycoprotein
290	MET	MET	Hepatocyte growth factor receptor
291	METRNL	METRNL	Meteorin-like protein
292	MFAP5	MFAP5	Microfibrillar-associated protein 5
293	MGMT	MGMT	Methylated-DNA–protein-cysteine methyltransferase
294	MILR1	MILR1	Allergin-1
295	MMP-1	MMP1	Matrix metalloproteinase-1
296	MMP-10	MMP10	Matrix metalloproteinase-10
297	MMP-2	MMP2	Matrix metalloproteinase-2
298	MMP-3	MMP3	Matrix metalloproteinase-3
299	MMP-9	MMP9	Matrix metalloproteinase-9
300	MPO	MPO	Myeloperoxidase
301	NADK	NADK	NAD kinase
302	NCAM1	NCAM1	Neural cell adhesion molecule 1
303	NCR1	NCR1	Natural cytotoxicity triggering receptor 1
304	NECTIN2	PVRL2	Nectin-2
305	NF2	NF2	Merlin
306	NFATC3	NFATC3	Nuclear factor of activated T-cells, cytoplasmic 3
307	NID1	NID1	Nidogen-1
308	NOMO1	NOMO1	Nodal modulator 1
309	Notch 3	NOTCH3	Neurogenic locus notch homolog protein 3
310	NOTCH1	NOTCH1	Neurogenic locus notch homolog protein 1
311	NPDC1	NPDC1	Neural proliferation differentiation and control protein 1
312	NPTXR	NPTXR	Neuronal pentraxin receptor
313	NQO2	NQO2	Ribosyldihydronicotinamide dehydrogenase [quinone]
314	NRP1	NRP1	Neuropilin-1
315	NRTN	NRTN	Neurturin
316	NT-3	NTF3	Neurotrophin-3
317	NT-proBNP	NPPB	N-terminal prohormone brain natriuretic peptide

No.	OLINK	STRING	Full name
318	NTF4	NTF4	Neurotrophin-4
319	OPG	TNFRSF11B	Osteoprotegerin
320	OPN	SPP1	Osteopontin
321	OSM	OSM	Oncostatin-M
322	OSMR	OSMR	Oncostatin-M-specific receptor subunit beta
323	PADI2	PADI2	Protein-arginine deiminase type-2
324	PAG1	PAG1	Phosphoprotein associated with glycosphingolipid-enriched microdomains 1
325	PAI	SERPINE1	Plasminogen activator inhibitor 1
326	PAM	PAM	Peptidyl-glycine alpha-amidating monooxygenase
327	PCOLCE	PCOLCE	Procollagen C-endopeptidase enhancer 1
328	PCSK9	PCSK9	Proprotein convertase subtilisin/kexin type 9
329	PD-L1	CD274	Programmed cell death 1 ligand 1
330	PDGF subunit A	PDGFRA	Platelet-derived growth factor subunit A
331	PECAM-1	PECAM1	Platelet endothelial cell adhesion molecule
332	PGLYRP1	PGLYRP1	Peptidoglycan recognition protein 1
333	PI3	PI3	Elafin
334	PIK3AP1	PIK3AP1	Phosphoinositide 3-kinase adapter protein 1
335	PILRB	PILRB	Paired immunoglobulin-like type 2 receptor beta
336	PLA2G7	PLA2G7	Platelet-activating factor acetylhydrolase
337	PLC	HSPG2	Perlecan
338	PLTP	PLTP	Phospholipid transfer protein
339	PLXNA4	PLXNA4	Plexin-A4
340	PLXNB2	PLXNB2	Plexin-B2
341	PON3	PON3	Paraoxonase
342	PPP1R2	PPP1R2	Protein phosphatase inhibitor 2
343	PPP1R9B	PPP1R9B	Neurabin-2
344	PRCP	PRCP	Lysosomal Pro-X carboxypeptidase
345	PRDX1	PRDX1	Peroxiredoxin-1
346	PRDX3	PRDX3	Thioredoxin-dependent peroxide reductase, mitochondrial
347	PRDX5	PRDX5	Peroxiredoxin-5, mitochondrial
348	PRKCQ	PRKCQ	Protein kinase C theta type
349	PROC	PROC	Vitamin K-dependent protein C
350	PRSS2	PRSS2	Trypsin-2
351	PRTN3	PRTN3	Myeloblastin
352	PSIP1	PSIP1	PC4 and SFRS1-interacting protein
353	PSP-D	SFTPD	Pulmonary surfactant-associated protein D
354	PTH1R	PTH1R	Parathyroid hormone/parathyroid hormone-related peptide receptor
355	PTPRS	PTPRS	Receptor-type tyrosine-protein phosphatase S
356	QDPR	QDPR	Dihydropteridine reductase
357	QPCT	QPCT	Glutaminyl-peptide cyclotransferase

No.	OLINK	STRING	Full name
358	RARRES2	RARRES2	Retinoic acid receptor responder protein 2
359	REG1A	REG1A	Lithostathine-1-alpha
360	REG3A	REG3A	Regenerating islet-derived protein 3-alpha
361	REG4	REG4	Regenerating islet-derived protein 4
362	RETN	RETN	Resistin
363	RNASE3	RNASE3	Eosinophil cationic protein
364	ROR1	ROR1	Inactive tyrosine-protein kinase transmembrane receptor ROR1
365	RTN4R	RTN4R	Reticulon-4 receptor
366	S100P	S100P	Protein S100-P
367	SAA4	SAA4	Serum amyloid A-4 protein
368	SCF	SCF	Stem cell factor
369	SCGB3A2	SCGB3A2	Secretoglobin family 3A member 2
370	SDC4	SDC4	Syndecan-4
371	SELE	SELE	E-selectin
372	SELL	SELL	L-selectin
373	SELP	SELP	P-selectin
374	SEMA3F	SEMA3F	Semaphorin-3F
375	SERPINA5	SERPINA5	Plasma serine protease inhibitor
376	SERPINA7	SERPINA7	Thyroxine-binding globulin
377	SERPINB6	SERPINB6	Serpin B6
378	SERPINB8	SERPINB8	Serpin B8
379	SH2B3	SH2B3	SH2B adapter protein 3
380	SH2D1A	SH2D1A	SH2 domain-containing protein 1A
381	SHPS-1	SIRPA	Tyrosine-protein phosphatase non-receptor type substrate 1
382	SIGLEC7	SIGLEC7	Sialic acid-binding Ig-like lectin 7
383	SIRT2	SIRT2	SIR2-like protein 2
384	SIT1	SIT1	Signaling threshold-regulating transmembrane adapter 1
385	SLAMF1	SLAMF1	Signaling lymphocytic activation molecule
386	SNAP23	SNAP23	Synaptosomal-associated protein 23
387	SOD1	SOD1	Superoxide dismutase [Cu-Zn]
388	SOST	SOST	Sclerostin
389	SPARCL1	SPARCL1	SPARC-like protein 1
390	SPON1	SPON1	Spondin-1
391	SPRY2	SPRY2	Protein sprouty homolog 2
392	SRPK2	SRPK2	SRSF protein kinase 2
393	SSC4D	SSC4D	Scavenger receptor cysteine-rich domain-containing group B protein
394	ST1A1	SULT1A1	Sulfotransferase 1A1
395	ST2	IL1RL1	ST2 protein
396	ST6GAL1	ST6GAL1	Beta-galactoside alpha-2,6-sialyltransferase 1

No.	OLINK	STRING	Full name
397	STAMBP	STAMBP	STAM-binding protein
398	STC1	STC1	Stanniocalcin-1
399	SUMF2	SUMF2	Sulfatase-modifying factor 2
400	t-PA	PLAT	Tissue-type plasminogen activator
401	TANK	TANK	TRAF family member-associated NF-kappa-B activator
402	TCN2	TCN2	Transcobalamin-2
403	TFF2	TFF2	Trefoil factor 2
404	TFF3	TFF3	Trefoil factor 3
405	TFPI	TFPI	Tissue factor pathway inhibitor
406	TGF-alpha	TGFA	Transforming growth factor alpha
407	TGFBI	TGFBI	Transforming growth factor-beta-induced protein ig-h3
408	TGFBR3	TGFBR3	Transforming growth factor beta receptor type 3
409	THBS4	THBS4	Thrombospondin-4
410	THOP1	THOP1	Thimet oligopeptidase
411	TIE1	TIE1	Tyrosine-protein kinase receptor Tie-1
412	TIMD4	TIMD4	T-cell immunoglobulin and mucin domain-containing protein 4
413	TIMP1	TIMP1	Metalloproteinase inhibitor 1
414	TIMP4	TIMP4	Metalloproteinase inhibitor 4
415	TINAGL1	TINAGL1	Tubulointerstitial nephritis antigen-like
416	TLT-2	TREML1	Trem-like transcript 2 protein
417	TNC	TNC	Tenascin
418	TNF	TNF	Tumor necrosis factor
419	TNF-R1	TNFRSF1A	Tumor necrosis factor receptor 1
420	TNF-R2	TNFRSF1B	Tumor necrosis factor receptor 2
421	TNFB	LTA	TNF-beta
422	TNFRSF10C	TNFRSF10C	Tumor necrosis factor receptor superfamily member 10C
423	TNFRSF14	TNFRSF14	Tumor necrosis factor receptor superfamily member 14
424	TNFRSF9	TNFRSF9	Tumor necrosis factor receptor superfamily member 9
425	TNFSF13B	TNFSF13B	Tumor necrosis factor ligand superfamily member 13B
426	TNFSF14	TNFSF14	Tumor necrosis factor ligand superfamily member 14
427	TNXB	TNXB	Tenascin-X
428	TPSAB1	TPSAB1	Tryptase alpha/beta-1
429	TR	TFRC	Transferrin receptor protein 1
430	TR-AP	ACP5	Tartrate-resistant acid phosphatase type 5
431	TRAF2	TRAF2	TNF receptor-associated factor 2
432	TRAIL	TNFSF10	TNF-related apoptosis-inducing ligand

No.	OLINK	STRING	Full name
433	TRANCE	TNFSF11	TNF-related activation-induced cytokine
434	TREM1	TREM1	Triggering receptor expressed on myeloid cells 1
435	TRIM21	TRIM21	E3 ubiquitin-protein ligase TRIM21
436	TRIM5	TRIM5	Tripartite motif-containing protein 5
437	TSHB	TSHB	Thyrotropin subunit beta
438	TSLP	TSLP	Thymic stromal lymphopoietin
439	TWEAK	TNFSF12	Tumor necrosis factor-like weak
440	TXNDC5	TXNDC5	Thioredoxin domain-containing protein 5
441	TYMP	TYMP	Thymidine phosphorylase
442	TYRO3	TYRO3	Tyrosine-protein kinase receptor TYRO3
443	U-PAR	PLAUR	Urokinase plasminogen activator surface receptor
444	UMOD	UMOD	Uromodulin
445	uPA	PLAU	Urokinase-type plasminogen activator
446	USP8	USP8	Ubiquitin carboxyl-terminal hydrolase 8
447	VASN	VASN	Vasorin
448	VCAM1	VCAM1	Vascular cell adhesion protein 1
449	VCAN	VCAN	Versican core protein
450	VEGF-A	VEGFA	Vascular endothelial growth factor A
451	vWF	VWF	von Willebrand factor
452	ZBTB16	ZBTB16	Zinc finger and BTB domain-containing protein 16
