# Supplementary material for: Proteomic machine-learning signatures associated with carotid plaque: A population-based hypothesis-generating study
Source: PLoS One. 2026 Sep 24;21(9):e0358765. doi: 10.1371/journal.pone.0358765 (PMC13606957; doi:10.1371/journal.pone.0358765)
Supplement: Supplementary file 2 [file pone.0358765.s002.pdf]

S2 Table: All important proteins in age-restricted group.

**Protein**	**Importance**	**Protein**	**Importance**	**Protein**	**Importance**
GDF15	19.8	SIGLEC7	5.9	MEPE	4.5
CDCP1	14.0	REG4	5.7	FBP1	4.3
CHIT1	12.2	ACP5	5.7	FABP4	4.2
FLT3LG	8.7	CXCL9	5.6	SELL	4.1
CDH2	8.5	NPPB	5.6	GAL	4.1
CRTAC1	8.1	CSTB	5.4	EGFR	4.0
TFPI	8.1	SERPINE1	5.3	OSMR	3.8
SOST	7.7	TFF2	4.9	IL7	3.7
TNFRSF11B	7.2	IGFBP7	4.8	APLP1	3.6
FAS	6.6	EFEMP1	4.8	TIMP4	3.6
LGALS4	6.4	LDLR	4.7	IL18R1	3.5
PLAT	6.4	LAMP3	4.7	SPON1	3.4
KIT	6.3	PDGFRA	4.6	CCL11	3.4
CR2	6.3	CA3	4.5	shadowMax	3.4
CTSD	6.0				
