# Supplementary material for: Proteomic machine-learning signatures associated with carotid plaque: A population-based hypothesis-generating study
Source: PLoS One. 2026 Sep 24;21(9):e0358765. doi: 10.1371/journal.pone.0358765 (PMC13606957; doi:10.1371/journal.pone.0358765)
Supplement: Supplementary file 3 [file pone.0358765.s003.docx]

1. Please ensure that your manuscript meets PLOS ONE's style requirements, including those for file naming. The PLOS ONE style templates can be found at

https://journals.plos.org/plosone/s/file?id=wjVg/PLOSOne_formatting_sample_main_body.pdf and

<https://journals.plos.org/plosone/s/file?id=ba62/PLOSOne_formatting_sample_title_authors_affiliations.pdf>.

**The manuscript was corrected according to journal style requirements. However, when using the LaTeX system, we did not always have control over the exact placement of figures and tables.**

2. The ethical approval number(s) listed in the manuscript and/or submission metadata does not match the approval number on the ethical approval document you provided. Please ensure that all approval numbers are correct.

**The incorrect number provided in the editorial system was corrected.**

3. Please note that PLOS One has specific guidelines on code sharing for submissions in which author-generated code underpins the findings in the manuscript. In these cases, all author-generated code must be made available without restrictions upon publication of the work. Please review our guidelines at https://journals.plos.org/plosone/s/materials-and-software-sharing#loc-sharing-code and ensure that your code is shared in a way that follows best practice and facilitates reproducibility and reuse.

**The code is available at Zenodo repository, according to PLOS One guidelines:**

<https://doi.org/10.5281/zenodo.21769048> **.**

4. Thank you for stating the following in the Acknowledgments Section of your manuscript:

“This study was supported by funds of the Ministry of Science and Higher education (Poland) within the project ”The Excellence Initiative - Research University” (proteomic research), and from statutory funds of Medical University of Bialystok. The funding source had no involvement in study design, data collection, analysis, and interpretation of data, writing of the report or decision to submit the article for publication. There were no article processing charges.”

We note that you have provided funding information that is not currently declared in your Funding Statement. However, funding information should not appear in the Acknowledgments section or other areas of your manuscript. We will only publish funding information present in the Funding Statement section of the online submission form.

Please remove any funding-related text from the manuscript and let us know how you would like to update your Funding Statement. Currently, your Funding Statement reads as follows:

“This study was supported by funds of the Ministry of Science and Higher education (Poland) within the project ”The Excellence Initiative - Research University” (proteomic research), and from statutory funds of Medical University of Bialystok. The funding source had no involvement in study design, data collection, analysis, and interpretation of data, writing of the report or decision to submit the article for publication. There were no article processing charges.”

Please include your amended statements within your cover letter; we will change the online submission form on your behalf.

**The funding section has been removed from the manuscript. The statement provided above is correct, except for a spelling error: 'Education' should be written in capital letters. This information was also included in the cover letter.**

5. Thank you for stating the following financial disclosure:

“This study was supported by funds of the Ministry of Science and Higher education (Poland) within the project ”The Excellence Initiative - Research University” (proteomic research), and from statutory funds of Medical University of Bialystok. The funding source had no involvement in study design, data collection, analysis, and interpretation of data, writing of the report or decision to submit the article for publication. There were no article processing charges.”

We note that one or more of the authors is affiliated with the funding organization, indicating the funder may have had some role in the design, data collection, analysis or preparation of your manuscript for publication; in other words, the funder played an indirect role through the participation of the co-authors. If the funding organization did not play a role in the study design, data collection and analysis, decision to publish, or preparation of the manuscript and only provided financial support in the form of authors' salaries and/or research materials, please do the following:

1. Review your statements relating to the author contributions, and ensure you have specifically and accurately indicated the role(s) that these authors had in your study. These amendments should be made in the online form.

2. Confirm in your cover letter that you agree with the following statement, and we will change the online submission form on your behalf:

“The funder provided support in the form of salaries for authors [insert relevant initials], but did not have any additional role in the study design, data collection and analysis, decision to publish, or preparation of the manuscript. The specific roles of these authors are articulated in the ‘author contributions’ section.

**We all agree with the following statement: “The funder provided support in the form of salaries for authors [A.S, K.A.K., U.R., M.D., Z.S., K.M.], but did not have any additional role in the study design, data collection and analysis, decision to publish, or preparation of the manuscript. The specific roles of these authors are articulated in the ‘author contributions’ section.”**

**We carefully reviewed statements relating to the author contributions, and ensured they were accurate. In the case of K.A.K. funding acquisition referred to external organizations only. This information was also included in the cover letter.**

6. Thank you for uploading your study's underlying data set. Unfortunately, the repository you have noted in your Data Availability statement does not qualify as an acceptable data repository according to PLOS's standards.

At this time, please upload the minimal data set necessary to replicate your study's findings to a stable, public repository (such as figshare or Dryad) and provide us with the relevant URLs, DOIs, or accession numbers that may be used to access these data. For a list of recommended repositories and additional information on PLOS standards for data deposition, please see <https://journals.plos.org/plosone/s/recommended-repositories>.

**The database has been deposited in the repository Zenodo repository:**

<https://zenodo.org/records/21769048>

<https://doi.org/10.5281/zenodo.21769048>

7. Your ethics statement should only appear in the Methods section of your manuscript. If your ethics statement is written in any section besides the Methods, please move it to the Methods section and delete it from any other section. Please ensure that your ethics statement is included in your manuscript, as the ethics statement entered into the online submission form will not be published alongside your manuscript.

**The ethics statement was moved to the Methods section.**

8. Please ensure that you refer to Figure 4 in your text as, if accepted, production will need this reference to link the reader to the figure.

**The numbering and citation of figures have been checked and corrected.**

9. We note you have included a table to which you do not refer in the text of your manuscript. Please ensure that you refer to Table 2 in your text; if accepted, production will need this reference to link the reader to the Table.

**The numbering and citation of tables have been checked and corrected.**

10. Please include captions for your Supporting Information files at the end of your manuscript, and update any in-text citations to match accordingly. Please see our Supporting Information guidelines for more information: <http://journals.plos.org/plosone/s/supporting-information>.

**Captions for the supporting information files have been added to the end of the manuscript and the in-text citations have been updated.**

11. If the reviewer comments include a recommendation to cite specific previously published works, please review and evaluate these publications to determine whether they are relevant and should be cited. There is no requirement to cite these works unless the editor has indicated otherwise.

**This does not apply to the reviews of our work.**

Reviewers' comments:

 Reviewer's Responses to Questions

**Comments to the Author**

 1. Is the manuscript technically sound, and do the data support the conclusions?

 The manuscript must describe a technically sound piece of scientific research with data that supports the conclusions. Experiments must have been conducted rigorously, with appropriate controls, replication, and sample sizes. The conclusions must be drawn appropriately based on the data presented.

Reviewer #1: Yes

Reviewer #2: Yes

2. Has the statistical analysis been performed appropriately and rigorously?

Reviewer #1: Yes

Reviewer #2: Yes

3. Have the authors made all data underlying the findings in their manuscript fully available?

 The [PLOS Data policy](http://track.editorialmanager.com/CL0/http:%2F%2Fwww.plosone.org%2Fstatic%2Fpolicies.action%23sharing/1/010f019ed34e20ed-f1b86860-6951-44a9-a485-25015f794d88-000000/RY8Z-0BXq4DV68RQW3kNttqZ5YbIRl3AF8sUET9fVog=258) requires authors to make all data underlying the findings described in their manuscript fully available without restriction, with rare exception (please refer to the Data Availability Statement in the manuscript PDF file). The data should be provided as part of the manuscript or its supporting information, or deposited to a public repository. For example, in addition to summary statistics, the data points behind means, medians and variance measures should be available. If there are restrictions on publicly sharing data—e.g. participant privacy or use of data from a third party—those must be specified.

Reviewer #1: Yes

Reviewer #2: No

4. Is the manuscript presented in an intelligible fashion and written in standard English?

 PLOS ONE does not copyedit accepted manuscripts, so the language in submitted articles must be clear, correct, and unambiguous. Any typographical or grammatical errors should be corrected at revision, so please note any specific errors here.

Reviewer #1: Yes

Reviewer #2: Yes

5. Review Comments to the Author

 Please use the space provided to explain your answers to the questions above. You may also include additional comments for the author, including concerns about dual publication, research ethics, or publication ethics. (Please upload your review as an attachment if it exceeds 20,000 characters)

Reviewer #1: Dear Authors,
 As I am not an expert in proteomics, or biomarker research, I focused this review on the study’s overall design, clarity, and clinical interpretability rather than the technical details.
 In my opinion, the manuscript is well-written. The objectives and methods are appropriately described, and the results are presented in a logical, easy-to-understand manner. The findings appear consistent with current knowledge regarding carotid plaque and seem clinically plausible. I believe this manuscript is suitable for publication pending minor revisions.
 My suggestions are as follows:
 1. Briefly comment on how the reported predictive performance compares with existing clinical risk scores.

**Thank you for this suggestion. We added the following paragraph to the discussion:**

**“To the best of our knowledge, there are currently no clinical risk scales in use for predicting the presence of CPs. In our study, we obtained a multivariate proteomic signature of this clinical condition. The risk scores used in practice relate to the 10-year risk of fatal and non-fatal cardiovascular disease events (SCORE2) [28] or the clinical likelihood of obstructive atherosclerotic coronary artery disease [29]. Risk scores such as the Carotid Artery Risk Score or Carotid Plaque Score are used to assess the risk of stroke in patients with CPs or CS [30,31].”**

2. Clearly state which mechanistic interpretations remain speculative and emphasize the need for external validation in an independent cohort.

**We modified the text to emphasize that our results do not allow for drawing causal conclusions and need external validation.**

**Abstract:**

**We identified 42 significant biomarkers associated with CP, the most important of which were GDF-15, CDCP1, CHIT1, FLT3LG, CDH2 and CRTAC1. Interestingly, the top 6 biomarkers had a highly speculative or unresolved association with atherosclerosis.**

**Next, we developed an ML-based CP fingerprint comprising 25 biomarkers, which showed good internal cross-validated discrimination in subjects aged 30–70 years. We also generated a network of interactions for the 42 biomarkers using STRING database modules, and performed a functional analysis to assess the biological significance of the interactions detected. These results are hypothesis-generating and require external, prospective validation to identify cause-and-effect relationships.**

**Aims: Additionally, we sought to analyse the network of associations between various biomarkers by employing STRING database algorithms. In our previous publication, we used routine statistical tests to address these issues and used only one panel of biomarkers (cardiometabolic) [ 3]. For this project, we used alternative advanced analytical tools to gain a deeper description ~~(understanding)~~ of the problem.**

**Conclusion:**

**In our study we developed an ML-based fingerprint of CP which showed good internal cross-validated discrimination in subjects aged 30–70 years and comprised 25 biomarkers. Its diagnostic odds ratio was comparable to a traditional clinical model (12.1 vs. 11.6). We identified 42 significant biomarkers associated with CP. The six most important proteins were: GDF-15, CDCP1, CHIT1, FLT3LG, CDH2 and CRTAC1. Among the biomarkers, GDF-15 had definitely the highest importance score. The potential association of the top biomarkers with atherosclerosis is highly speculative**

**and only hypothesis-generating. … Our findings require validation in prospective studies, as well as in different cohorts with different genetic backgrounds, lifestyles, and environments. They do not permit the identification of cause-and-effect relationships. Nevertheless, they are worth considering for further mechanistic studies.**

Reviewer #2: Recommendation: Major revision, with serious methodological concerns

 Although the topic is relevant and the dataset is potentially valuable, the current manuscript has substantial methodological and interpretational weaknesses. The main concerns are possible information leakage in biomarker filtering and feature selection, insufficient validation, overstatement of mechanistic conclusions, lack of calibration and clinical utility assessment, and inadequate reporting of the machine-learning pipeline. In its current form, the manuscript does not provide sufficiently robust evidence to support claims of “excellent” prediction or molecular mechanism discovery.

 Major critical comments
 1. Possible information leakage may invalidate the reported model performance

 The authors state that biomarkers were excluded when more than 25% of samples were below LOD and the biomarker level was not a differentiating factor between groups. If this filtering step was performed using the full dataset before cross-validation, then information from the outcome was used before model validation. This would constitute data leakage and could substantially inflate the reported AUC and feature importance. The authors must clearly state whether all filtering, preprocessing, LOD handling, imputation, normalization, feature selection, and model building were performed strictly within each training fold. If not, the entire modelling pipeline should be repeated using fully nested cross-validation.

**Thank you for your comment. There is a practice to exclude biomarkers with a high percentage of samples with measurements below the LOD from the analysis, which can result in data leakage and overestimation of models. In our study, after consulting our machine learning experts, we decided to keep the data in the analysed database. Information stating that the biomarkers had been excluded was incorrect and has been removed from the manuscript. We also removed a fragment about the determination of LOD. Instead, we added information in the Methods:**

**“We decided not to remove from the analysis biomarkers with a high percentage of samples with measurements below the LOD in order to avoid data leakage and overestimation of models. The proteomic data were not subjected to filtering or preprocessing. Data median imputation, normalization, feature selection, and model building were performed within each training fold.”**

2. The study is too dependent on age, and the proteomic signal may largely reflect age

 The authors report that all individuals younger than 30 years had no carotid plaque and all individuals older than 70 years had plaque; therefore, they restricted the analysis to participants aged 30–70 years. Even after this restriction, age remained by far the most important predictor. The RAFS results show that age alone achieved AUC around 0.782, and adding several proteins only modestly increased AUC. This raises a serious concern that many proteomic markers may be acting as age proxies rather than plaque-specific biomarkers. The authors should provide age-adjusted biomarker analyses, age-residualized protein models, stratified analyses by age groups, and a direct comparison with an age-only model.

**Atherosclerosis is strongly associated with age. Likewise, the levels of proteins associated with the presence of atherosclerotic plaques are also related to age. However, we present that these proteins also provide information beyond that explained by age alone. To support this, we first developed a regression model to predict age from proteomic data. We then constructed separate atherosclerosis prediction models for four age groups. All of these models achieved predictive performance above chance, suggesting that proteomic data contain information relevant to atherosclerosis that is not solely attributable to age. It should be noted, however, that the youngest and oldest age groups were poorly balanced, which may have affected model performance.**

**We added the following paragraph to the manuscript:**

**“To investigate the associations between proteins and age, two types of analyses were performed. First, age was predicted from proteomic data in a cross-validation loop. The resulting model achieved a Pearson correlation coefficient squared (R2) of 0.663 and a root mean squared error (RMSE) equal to 7.14. Feature selection revealed that many of the proteins selected for plaque prediction also contributed to age prediction (e.g. GDF-15, CDCP1, SOST, CXCL9, FLt3L). Next, separate proteomics-based models for atherosclerosis were developed for four age groups (30–39, 40–49, 50–59, and 60–70 years). For the 30–39-year age group, the model achieved an AUC of 0.630 (95% CI: 0.605–0.655).For the 40–49-year group, the AUC was 0.600 (95% CI: 0.579–0.621). The corresponding AUCs were 0.608 (95% CI: 0.583–0.632) for the 50–59-year group and 0.607 (95% CI: 0.578–0.636) for the 60–70-year group. There was a moderate correlation between age and proteome. Despite this, the predictive values of the models were comparable in all age groups (the AUC was lower than in the whole study group, probably due to smaller sample sizes).”**

**We do not present the calibration metrics for these models in the manuscript, as this is not the main focus of our analysis.**

3. The incremental value of proteomics is modest and overstated

 The traditional risk-factor model reportedly achieved AUC = 0.823, while the proteomic-only model achieved AUC = 0.816 and the combined model achieved AUC = 0.844. This improvement is numerically small. The authors repeatedly describe the model performance as “excellent,” but they do not show whether the improvement over clinical predictors is statistically significant or clinically meaningful. The authors should report ΔAUC with

confidence intervals, likelihood-ratio comparisons where applicable, Brier score, calibration slope, calibration intercept, net reclassification improvement, integrated discrimination improvement, and decision-curve analysis.

**We calculated the AUC with 95% confidence intervals, the Brier score, calibration slope, and calibration intercept for all models (Table 2). In addition, we generated calibration plots (Fig 3), observed-versus-predicted risk plots across risk deciles (Fig 4), and decision curve analysis (DCA) plots (Fig 5). The results and discussion of the net reclassification improvement (NRI) and integrated discrimination improvement (IDI) analyses are provided in the Supplementary Materials.**

 4. The manuscript contains a serious inconsistency in model numbering

 In the Results section, the authors state that the traditional risk-factor model achieved AUC = 0.823 and refer to “Table 4, model M5.” However, Table 4 defines M1 as the traditional risk-factor model and M5 as traditional risk factors plus an artificial variable based on proteins. This is not a minor typographical issue because the model comparisons are central to the paper. The authors must carefully check all model labels, table references, and interpretation of results.

**The numbering and citation of figures and tables have been checked and corrected. We also changed the model names to more generic ones.**

 5. Cross-validation alone is insufficient for the strength of the claims

 The study uses repeated 5-fold cross-validation, but there is no external validation. Since all participants come from a single cohort in one city, the generalizability of the model is unknown. The conclusion that a proteomic model has excellent predictive ability is premature. At minimum, the authors should state that the model has only internal validation and that its performance may not generalize to other populations, ethnic groups, age distributions, laboratory platforms, or clinical settings.

**We fully agree with the Reviewer that the lack of external validation constitutes a significant limitation of the study. This was addressed in the conclusions and study limitations; however, it should indeed be emphasized and included in the abstract as well to ensure a reliable interpretation of the study. The manuscript has been revised in accordance with the Reviewer's recommendations.**

**Below are excerpts from the conclusions:**

**“In our study we developed an ML-based fingerprint of CP which showed good internal cross-validated discrimination in subjects aged 30–70 years and comprised 25 biomarkers. Its diagnostic odds ratio was comparable to a traditional clinical model (12.1 vs. 11.6). …**

**Our findings require validation in prospective studies, as well as in different cohorts with different genetic backgrounds, lifestyles, and environments. They do not permit the identification of cause-and-effect relationships. Nevertheless, they are worth considering for further mechanistic studies.”**

6. The title is misleading and should be changed

 The title says that machine learning helps to understand molecular mechanisms of carotid plaques. This is too strong. The study is cross-sectional, predictive, and hypothesis-generating. It does not establish molecular mechanisms. STRING analysis is based on existing database links, text mining, co-expression, and pathway annotations; it cannot demonstrate causal biology. A more accurate title would be:

 Machine learning identifies proteomic biomarkers associated with carotid plaque in a population-based cohort

 or

 Proteomic machine-learning signatures associated with carotid plaque: a population-based hypothesis-generating study.

**Thank you for the suggestion. We concluded that the second proposal constitutes the optimal title for the manuscript.**

7. The mechanistic interpretation is overextended

 The authors state that the top biomarkers may indicate novel pathomechanisms of atherosclerosis, but they also acknowledge that the association of the top six biomarkers with atherosclerosis is speculative or unresolved. This inconsistency should be corrected. The conclusion should be more cautious and should state that the findings generate hypotheses requiring biological and prospective validation.

**We agree with the Reviewer's comments and have reformulated the conclusions and abstract in accordance with the suggestions.**

**Abstract:” …Next, we developed an ML-based CP fingerprint comprising 25 biomarkers, which showed good internal cross-validated discrimination in subjects aged 30–70 years. “**

**“These results are hypothesis-generating and require external, prospective validation to identify cause-and-effect relationships.”**

**Conclusion:**

**“In our study we developed an ML-based fingerprint of CP which showed good internal cross-validated discrimination in subjects aged 30–70 years and comprised 25 biomarkers. Its diagnostic odds ratio was comparable to a traditional clinical model (12.1 vs. 11.6). We identified 42 significant biomarkers associated with CP. The six most important proteins were: GDF-15, CDCP1, CHIT1, FLT3LG, CDH2 and CRTAC1. Among the biomarkers, GDF-15 had definitely the highest importance score. The potential association of the top biomarkers with atherosclerosis is highly speculative**

**and only hypothesis-generating. … Our findings require validation in prospective studies, as well as in different cohorts with different genetic backgrounds, lifestyles, and environments. They do not permit the identification of cause-and-effect relationships. Nevertheless, they are worth considering for further mechanistic studies.”**

8. The use of Boruta does not establish independent biomarker effects

 Boruta identifies variables that improve Random Forest prediction, but this does not mean that the biomarkers are independently associated with carotid plaque. This is especially important in proteomics, where many markers are correlated with each other and with age, inflammation, renal function, metabolic status, and cardiovascular risk. The authors should not interpret Boruta importance as biological importance. They should provide adjusted regression models or other interpretable analyses for the top biomarkers, at least adjusting for age, sex, hypertension, diabetes, hypercholesterolemia, smoking, and obesity.

**We agree 100% with the Reviewer that Boruta importance should not be interpreted as biological importance by itself. The importance is based just on the utility of the biomarker for Random Forest models. This utility may stem from the direct involvement in the biological process under scrutiny but it may very well arise due to correlations with other effects.**

**Therefore, the relevance of the marker indicated by Boruta may be used to form a hypothesis about biological relevance or function, but not as proof of either.**

**However, we respectfully disagree that adjusted regression analyses of individual biomarkers are necessary to support the objectives of this study. Such analyses would address a different question: whether each protein is associated with CP independently of age and conventional risk factors. Our objective was instead to identify a multivariate proteomic signature, including correlated and partially redundant variables that jointly contribute to prediction. This distinction is important both biologically and methodologically, particularly because age-related processes are broadly reflected in the proteome and Random Forest can use multiple correlated variables to recover a robust predictive signal from individually noisy measurements.**

**This rationale is also directly relevant to the Reviewer’s following comment concerning the selection of the 25-protein panel. The choice of 25 proteins was an arbitrary but pragmatic decision, intended to retain a broad yet still interpretable signature. The 25 highest-ranked proteins were retained as a pragmatic, deliberately broad signature, whereas RAFS was used separately to identify a minimal-optimal set. This choice was also supported by the sensitivity of Boruta to sample size: in the cross-validation training sets containing 80% of the observations, it confirmed approximately one-third fewer variables than in the complete dataset. Retaining a broader panel therefore reduces dependence on a particular sample and provides Random Forest with multiple representations of the predictive signal.**

**To make the scope and interpretation of our analysis explicit, we introduced the following explanation in the Discussion:**

**Importantly, our aim was not to identify proteins associated with CP independently of age and conventional risk factors, but to identify a multivariate proteomic signature.**

**Because age was by far the strongest predictor of plaque status in our analyses, its relationship with the proteomic signature requires particular consideration.**

**Age is associated with numerous biological processes, and many age-related proteomic changes may therefore correlate with plaque status simply because both vary with age.**

**Still, at least some of these proteomic signals can be expected to reflect biological processes associated with plaque formation beyond their shared relationship with chronological age.**

**Since we cannot identify these processes and the related proteins a priori, we deliberately seek a broad multivariate signature rather than a small set of independently associated markers.**

**For this reason, we selected the 25 proteins with the highest Boruta importance scores rather than restricting the analysis to the minimal-optimal RAFS set.**

**This pragmatic cutoff retained a broader panel and reduced dependence on the particular correlated proteins selected in a given sample, especially because substantially fewer variables were confirmed in the smaller cross-validation training sets.**

**The selected proteins are therefore interpreted jointly as a hypothesis-generating signature, not as independently associated or causally implicated biomarkers.**

**This approach also improves robustness.**

**Random Forest combines information from multiple correlated variables and averages their predictive signal, reducing the impact of biological and measurement variability in individual proteins.**

9. The final 25-biomarker model is not sufficiently justified

 The abstract says the final ML model included 25 biomarkers, but the Methods describe RAFS and model comparisons with different feature sets. The rationale for selecting exactly 25 biomarkers is unclear. Was this based on Boruta importance, RAFS, cross-validated performance, biological interpretability, or arbitrary ranking? The authors should provide a transparent feature-selection flowchart and selection frequencies across all cross-validation folds.

**We thank the Reviewer for noting that the rationale for the 25-protein panel was not sufficiently clear. The panel consists of the 25 proteins with the highest Boruta importance scores obtained on the entire dataset. The number 25 was chosen pragmatically to retain a broad proteomic signature and was not derived from RAFS optimisation. RAFS was used separately to identify a minimal-optimal predictive set.**

10. The authors should report model uncertainty more rigorously

 The AUC values are reported with very small variability, for example ±0.004. It is unclear whether these are standard deviations across repeated folds, standard errors, or confidence intervals. These values may underestimate uncertainty because repeated cross-validation folds are not independent. The authors should provide proper 95% confidence intervals using appropriate resampling or bootstrap methods.

**Following the reviewer's suggestion, we now report 95% confidence intervals for all models (see Table 2). The confidence intervals were estimated using the bootstrap method with 2,000 resamples.**

11. Calibration is completely missing

 AUC alone is insufficient for evaluating a clinical prediction model. A model can have acceptable discrimination but poor calibration. The authors should provide calibration plots, calibration-in-the-large, calibration slope, Brier score, and observed-versus-predicted risk across risk deciles. Without calibration, the model cannot be interpreted as clinically useful.

**Thank you for the suggestion. We calculated confidence intervals, Brier score, calibration scope and calibration intercepts (all results are in Table 2). We also made calibration plots (see Fig 3) and observed-versus-predicted risk across risk deciles (see Fig 4).**

12. Threshold-dependent metrics are not interpretable without the threshold

 The authors report accuracy, precision, sensitivity, MCC, and diagnostic odds ratio, but they do not clearly specify the probability threshold used to classify participants as plaque-positive or plaque-negative. Was it 0.5, Youden index, class prevalence, or another threshold? Without this information, these metrics are not reproducible and may be misleading.

**Thank you for this valuable remark. We initially used the standard classification threshold of 0.5. In addition, we calculated all performance metrics using thresholds determined by the Youden index and by the class prevalence. The corresponding results are presented in Table 3.**

 13. The claim that traditional risk factors are less important than commonly believed is not supported

 The manuscript states that the role of traditional risk factors is small and less than commonly believed. This is too strong and potentially misleading. The authors used simplified binary definitions for hypertension, diabetes, hypercholesterolemia, obesity, and smoking. Continuous measures such as systolic blood pressure, LDL-C, HbA1c, pack-years, kidney function, and medication use may have stronger predictive value. The apparent weakness of traditional risk factors may reflect crude measurement, not true biological irrelevance.

**Our study was not designed to undermine the importance of traditional risk factors in atherosclerosis. Therefore, we have removed the aforementioned sentence. Additionally, we prepared models Mext and Mextprot including the suggested continuous variables, which had comparable AUC and markedly better calibration measures than the binary ones. However, given the limitations of our design, we should not jump to conclusions.**

**We have added the following excerpt to the discussion:**

**In our study, the model incorporating risk factors as continuous variables did not demonstrate better predictive value than the model using binary variables. However, the calibration measures were markedly better. Due to the limitations of our study design, we cannot draw any causal conclusions; we can only infer the utility of the panels as a CP fingerprint.**

14. Baseline clinical characteristics are insufficient

 The authors refer readers to a previous publication for detailed clinical characteristics. This is not adequate. This manuscript must include a complete baseline table for the analyzed N=548 subgroup, stratified by carotid plaque status. The table should include age, sex, BMI, blood pressure, lipid measures, HbA1c/glucose, smoking, diabetes, hypertension, hypercholesterolemia, medications, and relevant laboratory markers.

**Table 1 presenting the general characteristics of the groups and incorporating the aforementioned variables, has been added to the manuscript.**

15. Missing data handling is unclear

 The manuscript does not adequately explain missing data handling. The authors used participants with complete carotid ultrasound and proteomic measurements, but it is unclear how many participants were excluded and whether excluded participants differed systematically from included participants. Complete-case analysis may introduce selection bias. A participant flow diagram is needed.

**The study group comprised 698 consecutive participants, all of whom underwent a complete carotid ultrasound. Proteomic measurements were missing for five subjects due to technical reasons in the laboratory. Proteomic data were missing for random participants; this was not due to clinical characteristics. The final analysed study group included 693 participants. As only one variable was missing, we did not prepare a dedicated flow diagram. However, we are ready to do so if required.**

**Below is the updated version of the manuscript regarding the results:**

**“The study group comprised 698 consecutive participants. Proteomic measurements were missing for five random subjects for technical reasons. The final analysed group included 693 participants... “**

16. The ultrasound assessment requires stronger reporting

 The outcome is carotid plaque presence, but the manuscript should report whether ultrasound operators were blinded to clinical/proteomic data, whether plaque assessment was standardized, and whether inter-observer or intra-observer reliability was assessed. Since the entire ML model depends on this binary outcome, outcome measurement reliability is essential.

**In the methods section, the description of standardization and reliability assessment of ultrasound examinations has been improved:**

**Plaque assessment was standardised based on the Mannheim Intima-Media “Thickness and Plaque Consensus [8 ], CP was defined as a local structure encroaching into the arterial lumen by at least 0.5 mm or 50% of the surrounding IMT, or having a thickness >1.5 mm measured from the media–adventitia interface to the intima–lumen interface. There were 3 ultrasound operators, they were blinded to clinical and proteomic data. Inter-rater reliability in the assessment of atherosclerotic plaques was evaluated in 100 individuals using Cohen’s kappa coefficient. High inter-rater reliability was demonstrated: between examiner 1 and 2 (κ = 0.786; 95% CI: 0.661–0.911; observed agreement: 90%; p < 0.001), between examiner 1 and 3 (κ = 0.696; 95% CI: 0.549–0.843; observed agreement: 86%; p < 0.001), and between examiner 2 and 3 (κ = 0.788; 95% CI: 0.665–0.911; observed agreement: 90%; p < 0.001). In addition, McNemar’s test did not reveal any significant systematic differences between the investigators in any of the comparisons (p = 0.344; p = 0.791; p = 0.754), indicating no significant asymmetry in the classification of the cases studied.”**

17. The study ignores plaque burden and plaque morphology

 The outcome is only presence/absence of carotid plaque. This is a crude endpoint. Plaque burden, number of plaques, location, stenosis degree, echogenicity, ulceration, and high-risk morphology would be more informative. The authors acknowledge that morphology was not studied, but this substantially limits the clinical and mechanistic interpretation.

**We strongly agree with this comment. This was already pointed out in the Limitations of the manuscript; in the version below, we developed this point. We find it very difficult to assess morphology of carotid plaques in the population study, but the other parameters, like plaque burden, number and location of plaques, can be the subject of further research.**

**“In this study, we only focused on the presence of CPs as a marker of cardiovascular risk. We did not study the morphology of CPs, which is a highly specialised procedure that should be performed by experienced physicians, not technicians. High-risk CP morphology has a strong documented association with stroke risk, regardless of the degree of stenosis.**

**However, an incorrect interpretation of atherosclerotic plaque morphology by unqualified personnel could lead to legal issues. The other parameters, like plaque burden, number and location of plaques, were not analysed, which limits clinical and mechanistic interpretation of our findings. The next limitation of our study is the cross-sectional design. The biomarkers we have identified as being associated with CPs should be validated in other populations, and their causative role in atherogenesis should be tested in prospective analyses. Given the aforementioned limitations, the study does not permit the identification of cause-and-effect relationships.”**

18. The biological network analysis is descriptive and should not drive mechanistic claims

 STRING analysis is useful for visualization, but it is not independent experimental evidence. Many links appear to be based on text mining or co-expression. The authors should report the STRING confidence score threshold, interaction sources, multiple-testing correction method, enrichment FDR, and MCL parameters. The manual clustering of biomarkers into biological functions should be supported by explicit criteria and references.

**We thank the Reviewer for this comment. It was never our intention to present the STRING analysis as independent experimental evidence or as validation of mechanistic claims. Its purpose was descriptive and hypothesis generating: to organize known and predicted associations, identify enriched biological processes, and guide subsequent literature-based interpretation. We appreciate that this distinction was not stated sufficiently explicitly in the original manuscript and have now clarified it throughout the revised text.**

**STRING functional enrichment results were used only as an initial guide for identifying potentially relevant biological processes. For each protein, we subsequently examined the database annotations accessible through STRING, including GeneCards, KEGG, GenBank, and UniProt, and reviewed the relevant original publications in PubMed. The final functional classes were assigned manually by the authors on the basis of this combined evidence. We have clarified this procedure and added further methodological details to the revised manuscript. The original version already cited publications supporting the functional interpretation of individual proteins and pathways, including references [24-27] among others. We have now added two further references [22-23].**

**We have also revised the legend of Fig 8 to clarify how the author-defined grouping is represented. Coloured sectors indicate the manually assigned functional classes, with the darker sector denoting the primary class. The six vertices of the hexagonal layout correspond to the six categories occurring as primary assignments, including the category for proteins not assigned to any functional class. Lipid regulation occurred only as a secondary assignment and therefore has no separate vertex. Edges connect proteins sharing at least one assigned functional class.**

**We have also added information on the STRING interaction settings, MCL parameters, and statistical criteria used for functional enrichment. STRING version 12.0 was used with a minimum required interaction score of 0.4 (medium confidence), all available interaction sources, and an MCL inflation parameter of 3. Functional enrichment terms were considered significant at FDR < 0.05 after Benjamini–Hochberg correction for multiple testing. The global PPI enrichment p-value was < 1 × 10⁻¹⁶.**

New version:

**In the first step, a network of interactions was created using all proteins identified as relevant by Boruta. STRING version 12.0 was used with a minimum required interaction score of 0.4 (medium confidence), and all available interaction sources were included. Clustering analysis of this network was performed using the Markov Clustering (MCL) algorithm [21], with the inflation parameter set to 3.**

**The STRING database also provides a functional enrichment analysis module, based on biological processes from the Gene Ontology database and KEGG pathways, among others. Functional enrichment terms were considered significant at an FDR below 0.05 after Benjamini--Hochberg correction for multiple testing.**

**In our study, we analysed the biological functions of the 25 biomarkers with the highest Boruta importance scores. The STRING functional enrichment results were used as an initial guide to the biological processes and pathways represented in this protein set. The protein descriptions and annotations available through STRING links to GeneCards, KEGG, GenBank and UniProt databases were subsequently reviewed.**

**Finally, relevant original publications were identified and reviewed in PubMed [22-27].**

**On the basis of this combined evidence, the proteins were manually assigned by the authors to functional groups relevant to atherogenesis. This manual functional grouping was independent of the MCL clustering of the STRING interaction network.

New version of the legend for Figure 8:
Functional grouping of the 25 most relevant markers. The node area is proportional to the Boruta importance score. Coloured sectors indicate functional classes manually assigned by the authors, with the darker sector denoting the primary class. The six vertices of the hexagonal layout correspond to the six categories occurring as primary assignments, including one category for proteins not assigned to any functional class. Lipid regulation occurred only as a secondary assignment and therefore has no separate vertex. Nodes are positioned according to their primary class, and edges connect proteins sharing at least one assigned functional class.**

19. Code availability is essential

 The Data Availability statement provides a DOI for the dataset, but the analysis code is not clearly provided. For a machine-learning manuscript, code availability is critical. The authors should provide all R scripts, preprocessing code, random seeds, package versions, model objects or model specifications, and instructions to reproduce Tables 1–4 and Figures 1–5.

**The analysis code has been provided, including R scripts, pre-processing code, random seeds, package versions, model objects, model specifications. Instructions on how to reproduce figures are also included. Tables were prepared manually based on results generated by R code.**

For this purpose we used Zenodo repository:

<https://doi.org/10.5281/zenodo.21769048> .

 20. The abstract overstates the findings

 The abstract states that the model had “excellent ability to predict CP” and that the top biomarkers may identify novel pathomechanisms. This should be toned down. The abstract should state that the model showed good internal cross-validated discrimination in an age-restricted subgroup and that the biological interpretation is hypothesis-generating.

**The abstract, discussion and conclusions have been toned down.The objectives of the study were also modified.**

**Abstract:” …Next, we developed an ML-based CP fingerprint comprising 25 biomarkers, which showed good internal cross-validated discrimination in subjects aged 30–70 years. “**

**“These results are hypothesis-generating and require external, prospective validation to identify cause-and-effect relationships.”**

**Conclusion:**

**“In our study we developed an ML-based fingerprint of CP which showed good internal cross-validated discrimination in subjects aged 30–70 years and comprised 25 biomarkers. Its diagnostic odds ratio was comparable to a traditional clinical model (12.1 vs. 11.6). We identified 42 significant biomarkers associated with CP. The six most important proteins were: GDF-15, CDCP1, CHIT1, FLT3LG, CDH2 and CRTAC1. Among the biomarkers, GDF-15 had definitely the highest importance score. The potential association of the top biomarkers with atherosclerosis is highly speculative**

**and only hypothesis-generating. … Our findings require validation in prospective studies, as well as in different cohorts with different genetic backgrounds, lifestyles, and environments. They do not permit the identification of cause-and-effect relationships. Nevertheless, they are worth considering for further mechanistic studies.”**

**Aims: Additionally, we sought to analyse the network of associations between various biomarkers by employing STRING database algorithms. In our previous publication, we used routine statistical tests to address these issues and used only one panel of biomarkers (cardiometabolic) [ 3]. For this project, we used alternative advanced analytical tools to gain a deeper description ~~(understanding)~~ of the problem.**

Minor but important comments
 CRTAC2 appears in the abstract, while CRTAC1 appears elsewhere. This inconsistency must be corrected.
 “excellent” is misspelled as “excelent.”
 “patomechanisms” should be corrected to “pathomechanisms.”
 “graf” should be corrected to “graph.”
 “Arterosclerosis” should be corrected to “Atherosclerosis.”
 “Abberivations” should be corrected to “Abbreviations.”
 P-values such as “< 2.2e↑16” appear incorrectly formatted and should likely be “< 2.2 × 10−16.”
 The authors should standardize biomarker names: GDF15, GDF-15, and GDF 15 are used inconsistently.
 The manuscript should avoid phrases such as “blood drop has excellent ability” unless supported by external validation and clinical utility analysis
 The conclusion should explicitly state that causality cannot be inferred.

**The above comments have been incorporated into the revised version of the manuscript. GDF-15 has been adopted as the standardized name.**

**The phrase above was changed to: These analyses show that proteomic data imprinted on a drop of blood have good internal cross-validated discrimination for predicting CP, in our dataset comparable to a traditional clinical model.**

**Additional changes:**

**The number of analyzed biomarkers was adjusted to 460 and the number of analyzed panels to 5 (the analysis included a cardiometabolic panel from the outset).**
