# Supplementary material for: Proteomic machine-learning signatures associated with carotid plaque: A population-based hypothesis-generating study
Source: PLoS One. 2026 Sep 24;21(9):e0358765. doi: 10.1371/journal.pone.0358765 (PMC13606957; doi:10.1371/journal.pone.0358765)
Supplement: Peer review history

# Peer review: Decision Letter 0

**DOI:** 10.1371/journal.pone.0358765.r001

**Authors:** Fahad Farhan Almutairi

16 Jun 2026

Dear Dr. Szpakowicz,

Thank you for submitting your manuscript to PLOS ONE. After careful consideration, we feel that it has merit but does not fully meet PLOS ONE’s publication criteria as it currently stands. Therefore, we invite you to submit a revised version of the manuscript that addresses the points raised during the review process.

Please submit your revised manuscript by Aug 15 2026 11:59PM. If you will need more time than this to complete your revisions, please reply to this message or contact the journal office at plosone@plos.org. When you're ready to submit your revision, log on to https://www.editorialmanager.com/pone/ and select the 'Submissions Needing Revision' folder to locate your manuscript file.

- A letter that responds to each point raised by the academic editor and reviewer(s). You should upload this letter as a separate file labeled 'Response to Reviewers'.
- A marked-up copy of your manuscript that highlights changes made to the original version. You should upload this as a separate file labeled 'Revised Manuscript with Track Changes'.
- An unmarked version of your revised paper without tracked changes. You should upload this as a separate file labeled 'Manuscript'.

If you would like to make changes to your financial disclosure, please include your updated statement in your cover letter. Guidelines for resubmitting your figure files are available below the reviewer comments at the end of this letter.

If applicable, we recommend that you deposit your laboratory protocols in protocols.io to enhance the reproducibility of your results. Protocols.io assigns your protocol its own identifier (DOI) so that it can be cited independently in the future. For instructions see: https://journals.plos.org/plosone/s/submission-guidelines#loc-laboratory-protocols. Additionally, PLOS ONE offers an option for publishing peer-reviewed Lab Protocol articles, which describe protocols hosted on protocols.io. Read more information on sharing protocols at https://plos.org/protocols?utm_medium=editorial-email&utm_source=authorletters&utm_campaign=protocols.

As the corresponding author, your ORCID iD is verified in the submission system and will appear in the published article. PLOS supports the use of ORCID, and we encourage all coauthors to register for an ORCID iD and use it as well. Please encourage your coauthors to verify their ORCID iD within the submission system before final acceptance, as unverified ORCID iDs will not appear in the published article. *Only*  the individual author can complete the verification step; PLOS staff *cannot*  verify ORCID iDs on behalf of authors.

We look forward to receiving your revised manuscript.

Kind regards,

Fahad Farhan Almutairi, PhD

Academic Editor

PLOS One

Journal Requirements:

When submitting your revision, we need you to address these additional requirements.

1. Please ensure that your manuscript meets PLOS ONE's style requirements, including those for file naming. The PLOS ONE style templates can be found at

https://journals.plos.org/plosone/s/file?id=wjVg/PLOSOne_formatting_sample_main_body.pdf and

https://journals.plos.org/plosone/s/file?id=ba62/PLOSOne_formatting_sample_title_authors_affiliations.pdf

2. The ethical approval number(s) listed in the manuscript and/or submission metadata does not match the approval number on the ethical approval document you provided. Please ensure that all approval numbers are correct.

3. Please note that PLOS One has specific guidelines on code sharing for submissions in which author-generated code underpins the findings in the manuscript. In these cases, all author-generated code must be made available without restrictions upon publication of the work. Please review our guidelines at https://journals.plos.org/plosone/s/materials-and-software-sharing#loc-sharing-code and ensure that your code is shared in a way that follows best practice and facilitates reproducibility and reuse.

4. Thank you for stating the following in the Acknowledgments Section of your manuscript:

“This study was supported by funds of the Ministry of Science and Higher education (Poland) within the project ”The Excellence Initiative - Research University” (proteomic research), and from statutory funds of Medical University of Bialystok. The funding source had no involvement in study design, data collection, analysis, and interpretation of data, writing of the report or decision to submit the article for publication. There were no article processing charges.”

We note that you have provided funding information that is not currently declared in your Funding Statement. However, funding information should not appear in the Acknowledgments section or other areas of your manuscript. We will only publish funding information present in the Funding Statement section of the online submission form.

Please remove any funding-related text from the manuscript and let us know how you would like to update your Funding Statement. Currently, your Funding Statement reads as follows:

“This study was supported by funds of the Ministry of Science and Higher education (Poland) within the project ”The Excellence Initiative - Research University” (proteomic research), and from statutory funds of Medical University of Bialystok. The funding source had no involvement in study design, data collection, analysis, and interpretation of data, writing of the report or decision to submit the article for publication. There were no article processing charges.”

Please include your amended statements within your cover letter; we will change the online submission form on your behalf.

5. Thank you for stating the following financial disclosure:

“This study was supported by funds of the Ministry of Science and Higher education (Poland) within the project ”The Excellence Initiative - Research University” (proteomic research), and from statutory funds of Medical University of Bialystok. The funding source had no involvement in study design, data collection, analysis, and interpretation of data, writing of the report or decision to submit the article for publication. There were no article processing charges.”

We note that one or more of the authors is affiliated with the funding organization, indicating the funder may have had some role in the design, data collection, analysis or preparation of your manuscript for publication; in other words, the funder played an indirect role through the participation of the co-authors. If the funding organization did not play a role in the study design, data collection and analysis, decision to publish, or preparation of the manuscript and only provided financial support in the form of authors' salaries and/or research materials, please do the following:

1. Review your statements relating to the author contributions, and ensure you have specifically and accurately indicated the role(s) that these authors had in your study. These amendments should be made in the online form.

2. Confirm in your cover letter that you agree with the following statement, and we will change the online submission form on your behalf:

“The funder provided support in the form of salaries for authors [insert relevant initials], but did not have any additional role in the study design, data collection and analysis, decision to publish, or preparation of the manuscript. The specific roles of these authors are articulated in the ‘author contributions’ section.

6. Thank you for uploading your study's underlying data set. Unfortunately, the repository you have noted in your Data Availability statement does not qualify as an acceptable data repository according to PLOS's standards.

At this time, please upload the minimal data set necessary to replicate your study's findings to a stable, public repository (such as figshare or Dryad) and provide us with the relevant URLs, DOIs, or accession numbers that may be used to access these data. For a list of recommended repositories and additional information on PLOS standards for data deposition, please see https://journals.plos.org/plosone/s/recommended-repositories.

7. Your ethics statement should only appear in the Methods section of your manuscript. If your ethics statement is written in any section besides the Methods, please move it to the Methods section and delete it from any other section. Please ensure that your ethics statement is included in your manuscript, as the ethics statement entered into the online submission form will not be published alongside your manuscript.

8. Please ensure that you refer to Figure 4 in your text as, if accepted, production will need this reference to link the reader to the figure.

9. We note you have included a table to which you do not refer in the text of your manuscript. Please ensure that you refer to Table 2 in your text; if accepted, production will need this reference to link the reader to the Table.

10. Please include captions for your Supporting Information files at the end of your manuscript, and update any in-text citations to match accordingly. Please see our Supporting Information guidelines for more information: http://journals.plos.org/plosone/s/supporting-information.

11. If the reviewer comments include a recommendation to cite specific previously published works, please review and evaluate these publications to determine whether they are relevant and should be cited. There is no requirement to cite these works unless the editor has indicated otherwise.

Reviewers' comments:

Reviewer's Responses to Questions

**Comments to the Author**

1. Is the manuscript technically sound, and do the data support the conclusions?

Reviewer #1: Yes

Reviewer #2: Yes

**********

2. Has the statistical analysis been performed appropriately and rigorously?

Reviewer #1: Yes

Reviewer #2: Yes

**********

3. Have the authors made all data underlying the findings in their manuscript fully available?

The PLOS Data policy

Reviewer #1: Yes

Reviewer #2: No

**********

4. Is the manuscript presented in an intelligible fashion and written in standard English?

Reviewer #1: Yes

Reviewer #2: Yes

**********

Reviewer #1: Dear Authors,

As I am not an expert in proteomics, or biomarker research, I focused this review on the study’s overall design, clarity, and clinical interpretability rather than the technical details.

In my opinion, the manuscript is well-written. The objectives and methods are appropriately described, and the results are presented in a logical, easy-to-understand manner. The findings appear consistent with current knowledge regarding carotid plaque and seem clinically plausible. I believe this manuscript is suitable for publication pending minor revisions.

My suggestions are as follows:

1. Briefly comment on how the reported predictive performance compares with existing clinical risk scores.

2. Clearly state which mechanistic interpretations remain speculative and emphasize the need for external validation in an independent cohort.

I have no further concerns.

Reviewer #2: Recommendation: Major revision, with serious methodological concerns

Although the topic is relevant and the dataset is potentially valuable, the current manuscript has substantial methodological and interpretational weaknesses. The main concerns are possible information leakage in biomarker filtering and feature selection, insufficient validation, overstatement of mechanistic conclusions, lack of calibration and clinical utility assessment, and inadequate reporting of the machine-learning pipeline. In its current form, the manuscript does not provide sufficiently robust evidence to support claims of “excellent” prediction or molecular mechanism discovery.

Major critical comments

1. Possible information leakage may invalidate the reported model performance

The authors state that biomarkers were excluded when more than 25% of samples were below LOD and the biomarker level was not a differentiating factor between groups. If this filtering step was performed using the full dataset before cross-validation, then information from the outcome was used before model validation. This would constitute data leakage and could substantially inflate the reported AUC and feature importance. The authors must clearly state whether all filtering, preprocessing, LOD handling, imputation, normalization, feature selection, and model building were performed strictly within each training fold. If not, the entire modelling pipeline should be repeated using fully nested cross-validation.

2. The study is too dependent on age, and the proteomic signal may largely reflect age

The authors report that all individuals younger than 30 years had no carotid plaque and all individuals older than 70 years had plaque; therefore, they restricted the analysis to participants aged 30–70 years. Even after this restriction, age remained by far the most important predictor. The RAFS results show that age alone achieved AUC around 0.782, and adding several proteins only modestly increased AUC. This raises a serious concern that many proteomic markers may be acting as age proxies rather than plaque-specific biomarkers. The authors should provide age-adjusted biomarker analyses, age-residualized protein models, stratified analyses by age groups, and a direct comparison with an age-only model.

3. The incremental value of proteomics is modest and overstated

The traditional risk-factor model reportedly achieved AUC = 0.823, while the proteomic-only model achieved AUC = 0.816 and the combined model achieved AUC = 0.844. This improvement is numerically small. The authors repeatedly describe the model performance as “excellent,” but they do not show whether the improvement over clinical predictors is statistically significant or clinically meaningful. The authors should report ΔAUC with confidence intervals, likelihood-ratio comparisons where applicable, Brier score, calibration slope, calibration intercept, net reclassification improvement, integrated discrimination improvement, and decision-curve analysis.

4. The manuscript contains a serious inconsistency in model numbering

In the Results section, the authors state that the traditional risk-factor model achieved AUC = 0.823 and refer to “Table 4, model M5.” However, Table 4 defines M1 as the traditional risk-factor model and M5 as traditional risk factors plus an artificial variable based on proteins. This is not a minor typographical issue because the model comparisons are central to the paper. The authors must carefully check all model labels, table references, and interpretation of results.

5. Cross-validation alone is insufficient for the strength of the claims

The study uses repeated 5-fold cross-validation, but there is no external validation. Since all participants come from a single cohort in one city, the generalizability of the model is unknown. The conclusion that a proteomic model has excellent predictive ability is premature. At minimum, the authors should state that the model has only internal validation and that its performance may not generalize to other populations, ethnic groups, age distributions, laboratory platforms, or clinical settings.

6. The title is misleading and should be changed

The title says that machine learning helps to understand molecular mechanisms of carotid plaques. This is too strong. The study is cross-sectional, predictive, and hypothesis-generating. It does not establish molecular mechanisms. STRING analysis is based on existing database links, text mining, co-expression, and pathway annotations; it cannot demonstrate causal biology. A more accurate title would be:

Machine learning identifies proteomic biomarkers associated with carotid plaque in a population-based cohort

or

Proteomic machine-learning signatures associated with carotid plaque: a population-based hypothesis-generating study

7. The mechanistic interpretation is overextended

The authors state that the top biomarkers may indicate novel pathomechanisms of atherosclerosis, but they also acknowledge that the association of the top six biomarkers with atherosclerosis is speculative or unresolved. This inconsistency should be corrected. The conclusion should be more cautious and should state that the findings generate hypotheses requiring biological and prospective validation.

8. The use of Boruta does not establish independent biomarker effects

Boruta identifies variables that improve Random Forest prediction, but this does not mean that the biomarkers are independently associated with carotid plaque. This is especially important in proteomics, where many markers are correlated with each other and with age, inflammation, renal function, metabolic status, and cardiovascular risk. The authors should not interpret Boruta importance as biological importance. They should provide adjusted regression models or other interpretable analyses for the top biomarkers, at least adjusting for age, sex, hypertension, diabetes, hypercholesterolemia, smoking, and obesity.

9. The final 25-biomarker model is not sufficiently justified

The abstract says the final ML model included 25 biomarkers, but the Methods describe RAFS and model comparisons with different feature sets. The rationale for selecting exactly 25 biomarkers is unclear. Was this based on Boruta importance, RAFS, cross-validated performance, biological interpretability, or arbitrary ranking? The authors should provide a transparent feature-selection flowchart and selection frequencies across all cross-validation folds.

10. The authors should report model uncertainty more rigorously

The AUC values are reported with very small variability, for example ±0.004. It is unclear whether these are standard deviations across repeated folds, standard errors, or confidence intervals. These values may underestimate uncertainty because repeated cross-validation folds are not independent. The authors should provide proper 95% confidence intervals using appropriate resampling or bootstrap methods.

11. Calibration is completely missing

AUC alone is insufficient for evaluating a clinical prediction model. A model can have acceptable discrimination but poor calibration. The authors should provide calibration plots, calibration-in-the-large, calibration slope, Brier score, and observed-versus-predicted risk across risk deciles. Without calibration, the model cannot be interpreted as clinically useful.

12. Threshold-dependent metrics are not interpretable without the threshold

The authors report accuracy, precision, sensitivity, MCC, and diagnostic odds ratio, but they do not clearly specify the probability threshold used to classify participants as plaque-positive or plaque-negative. Was it 0.5, Youden index, class prevalence, or another threshold? Without this information, these metrics are not reproducible and may be misleading.

13. The claim that traditional risk factors are less important than commonly believed is not supported

The manuscript states that the role of traditional risk factors is small and less than commonly believed. This is too strong and potentially misleading. The authors used simplified binary definitions for hypertension, diabetes, hypercholesterolemia, obesity, and smoking. Continuous measures such as systolic blood pressure, LDL-C, HbA1c, pack-years, kidney function, and medication use may have stronger predictive value. The apparent weakness of traditional risk factors may reflect crude measurement, not true biological irrelevance.

14. Baseline clinical characteristics are insufficient

The authors refer readers to a previous publication for detailed clinical characteristics. This is not adequate. This manuscript must include a complete baseline table for the analyzed N=548 subgroup, stratified by carotid plaque status. The table should include age, sex, BMI, blood pressure, lipid measures, HbA1c/glucose, smoking, diabetes, hypertension, hypercholesterolemia, medications, and relevant laboratory markers.

15. Missing data handling is unclear

The manuscript does not adequately explain missing data handling. The authors used participants with complete carotid ultrasound and proteomic measurements, but it is unclear how many participants were excluded and whether excluded participants differed systematically from included participants. Complete-case analysis may introduce selection bias. A participant flow diagram is needed.

16. The ultrasound assessment requires stronger reporting

The outcome is carotid plaque presence, but the manuscript should report whether ultrasound operators were blinded to clinical/proteomic data, whether plaque assessment was standardized, and whether inter-observer or intra-observer reliability was assessed. Since the entire ML model depends on this binary outcome, outcome measurement reliability is essential.

17. The study ignores plaque burden and plaque morphology

The outcome is only presence/absence of carotid plaque. This is a crude endpoint. Plaque burden, number of plaques, location, stenosis degree, echogenicity, ulceration, and high-risk morphology would be more informative. The authors acknowledge that morphology was not studied, but this substantially limits the clinical and mechanistic interpretation.

18. The biological network analysis is descriptive and should not drive mechanistic claims

STRING analysis is useful for visualization, but it is not independent experimental evidence. Many links appear to be based on text mining or co-expression. The authors should report the STRING confidence score threshold, interaction sources, multiple-testing correction method, enrichment FDR, and MCL parameters. The manual clustering of biomarkers into biological functions should be supported by explicit criteria and references.

19. Code availability is essential

The Data Availability statement provides a DOI for the dataset, but the analysis code is not clearly provided. For a machine-learning manuscript, code availability is critical. The authors should provide all R scripts, preprocessing code, random seeds, package versions, model objects or model specifications, and instructions to reproduce Tables 1–4 and Figures 1–5.

20. The abstract overstates the findings

The abstract states that the model had “excellent ability to predict CP” and that the top biomarkers may identify novel pathomechanisms. This should be toned down. The abstract should state that the model showed good internal cross-validated discrimination in an age-restricted subgroup and that the biological interpretation is hypothesis-generating.

Minor but important comments

CRTAC2 appears in the abstract, while CRTAC1 appears elsewhere. This inconsistency must be corrected.

“excellent” is misspelled as “excelent.”

“patomechanisms” should be corrected to “pathomechanisms.”

“graf” should be corrected to “graph.”

“Arterosclerosis” should be corrected to “Atherosclerosis.”

“Abberivations” should be corrected to “Abbreviations.”

P-values such as “< 2.2e↑16” appear incorrectly formatted and should likely be “< 2.2 × 10−16.”

The authors should standardize biomarker names: GDF15, GDF-15, and GDF 15 are used inconsistently.

The manuscript should avoid phrases such as “blood drop has excellent ability” unless supported by external validation and clinical utility analysis.

The conclusion should explicitly state that causality cannot be inferred.

**********

what does this mean?). If published, this will include your full peer review and any attached files.

If you choose “no”, your identity will remain anonymous but your review may still be made public.

**Do you want your identity to be public for this peer review?**  For information about this choice, including consent withdrawal, please see our Privacy Policy

Reviewer #1: **Yes:**  Takashi Kamio

Reviewer #2: **Yes:**  Leili Tapak

**********

[NOTE: If reviewer comments were submitted as an attachment file, they will be attached to this email and accessible via the submission site. Please log into your account, locate the manuscript record, and check for the action link "View Attachments". If this link does not appear, there are no attachment files.]

To ensure your figures meet our technical requirements, please review our figure guidelines: https://journals.plos.org/plosone/s/figures

You may also use PLOS’s free figure tool, NAAS, to help you prepare publication quality figures: https://journals.plos.org/plosone/s/figures#loc-tools-for-figure-preparation.

NAAS will assess whether your figures meet our technical requirements by comparing each figure against our figure specifications.

# Author comment: Author response to Decision Letter 1

**DOI:** 10.1371/journal.pone.0358765.r002

24 Aug 2026

Attached in the Resopnse to Reviewers document.

Attachment Submitted filename: Response to Reviewers.docx

# Editor report: Decision Letter 1

**DOI:** 10.1371/journal.pone.0358765.r003

**Authors:** Fahad Almutairi

7 Sep 2026

Proteomic machine-learning signatures associated with carotid plaque: a population-based hypothesis-generating study.

PONE-D-26-22059R1

Dear Dr. Szpakowicz,

We’re pleased to inform you that your manuscript has been judged scientifically suitable for publication and will be formally accepted for publication once it meets all outstanding technical requirements.

Within one week, you’ll receive an e-mail detailing the required amendments. When these have been addressed, you’ll receive a formal acceptance letter and your manuscript will be scheduled for publication.

An invoice will be generated when your article is formally accepted. Please note, if your institution has a publishing partnership with PLOS and your article meets the relevant criteria, all or part of your publication costs will be covered. Please make sure your user information is up-to-date by logging into Editorial Manager at Editorial Manager® and clicking the ‘Update My Information' link at the top of the page. For questions related to billing, please contact billing support.

If your institution or institutions have a press office, please notify them about your upcoming paper to help maximize its impact. If they’ll be preparing press materials, please inform our press team as soon as possible -- no later than 48 hours after receiving the formal acceptance. Your manuscript will remain under strict press embargo until 2 pm Eastern Time on the date of publication. For more information, please contact onepress@plos.org.

Kind regards,

Fahad Farhan Almutairi, PhD

Academic Editor

PLOS One

# Editor report: Acceptance letter

**DOI:** 10.1371/journal.pone.0358765.r004

**Authors:** Fahad Almutairi

PONE-D-26-22059R1

PLOS One

Dear Dr. Szpakowicz,

I'm pleased to inform you that your manuscript has been deemed suitable for publication in PLOS One. Congratulations! Your manuscript is now being handed over to our production team.

At this stage, our production department will prepare your paper for publication. This includes ensuring the following:

* All references, tables, and figures are properly cited

* All relevant supporting information is included in the manuscript submission,

* There are no issues that prevent the paper from being properly typeset

You will receive further instructions from the production team, including instructions on how to review your proof when it is ready. Please keep in mind that we are working through a large volume of accepted articles, so please give us a few days to review your paper and let you know the next and final steps.

Lastly, if your institution or institutions have a press office, please let them know about your upcoming paper now to help maximize its impact. If they'll be preparing press materials, please inform our press team within the next 48 hours. Your manuscript will remain under strict press embargo until 2 pm Eastern Time on the date of publication. For more information, please contact onepress@plos.org.

You will receive an invoice from PLOS for your publication fee after your manuscript has reached the completed accept phase. If you receive an email requesting payment before acceptance or for any other service, this may be a phishing scheme. Learn how to identify phishing emails and protect your accounts at https://explore.plos.org/phishing.

If we can help with anything else, please email us at customercare@plos.org.

Thank you for submitting your work to PLOS One and supporting open access.

Kind regards,

PLOS One Editorial Office Staff

on behalf of

Dr. Fahad Farhan Almutairi

Academic Editor

PLOS One
